# Discovery and characterization of the evolution, variation and functions of diversity-generating retroelements using thousands of genomes and metagenomes

**DOI:** 10.1186/s12864-019-5951-3

**Published:** 2019-07-19

**Authors:** Fazhe Yan, Xuelin Yu, Zhongqu Duan, Jinyuan Lu, Ben Jia, Yuyang Qiao, Chen Sun, Chaochun Wei

**Affiliations:** 1 School of Life Sciences and Biotechnology, Shanghai Jiao Tong University, 800 Dongchuan Road, Shanghai, 200240 China; 2 Shanghai Center for Bioinformation Technology, 1278 Keyuan Road, Pudong District, Shanghai, 201203 China

**Keywords:** DGR, Metagenome, Cassette structure, DGR sequence diversity, Adenine-specific mutations, Target gene

## Abstract

**Background:**

Diversity-generating retroelements (DGRs) are a unique family of retroelements that generate sequence diversity of DNA to benefit their hosts by introducing variations and accelerating the evolution of target proteins. They exist widely in bacteria, archaea, phage and plasmid. However, our understanding about DGRs in natural environments was still very limited.

**Results:**

We developed an efficient computational algorithm to identify DGRs, and applied it to characterize DGRs in more than 80,000 sequenced bacterial genomes as well as more than 4,000 human metagenome datasets. In total, we identified 948 non-redundant DGRs, which expanded the number of known DGRs in bacterial genomes and human microbiomes by about 55%, and provided a much more comprehensive reference for the study of DGRs. Phylogenetic analysis was done for identified DGRs. The putative target genes of DGRs were searched, and the functions of these target genes were investigated with a comprehensive alignment against the nr database.

**Conclusions:**

DGR system is a powerful and universal mechanism to generate diversity. DGR evolution is closely associated with the living environment and their cassette structures. Furthermore, it may impact a wide range of functional processes in addition to receptor-binding. These results significantly improved our understanding about DGRs.

**Electronic supplementary material:**

The online version of this article (10.1186/s12864-019-5951-3) contains supplementary material, which is available to authorized users.

## Background

DGRs are a unique family of retroelements that generate sequence diversity of DNA. They exist widely in bacteria, archaea, phage and plasmid, and benefit their hosts by introducing variations and accelerating the evolution of target proteins [1–5]. The first DGR was discovered in a Bordetella phage, BPP-1. Bordetella causes the respiratory infection in humans and many other mammals, controlled by the BvgAS signal transduction system [6]. The surface of Bordetella is highly variable owing to the dynamic gene expression in the infectious cycle [6]. The invasion of BPP-1 to Bordeltella relies on the phage tail fiber protein Mtd [7, 8]. With the process of mutagenic reverse transcription and cDNA integration, DGR introduces multiple nucleotide substitutions to Mtd gene and generates different receptor-binding molecules, thus making BPP-1 the ability to invade Bordetellae with diverse cell surfaces [2, 3, 9].

**Fig. 1:**
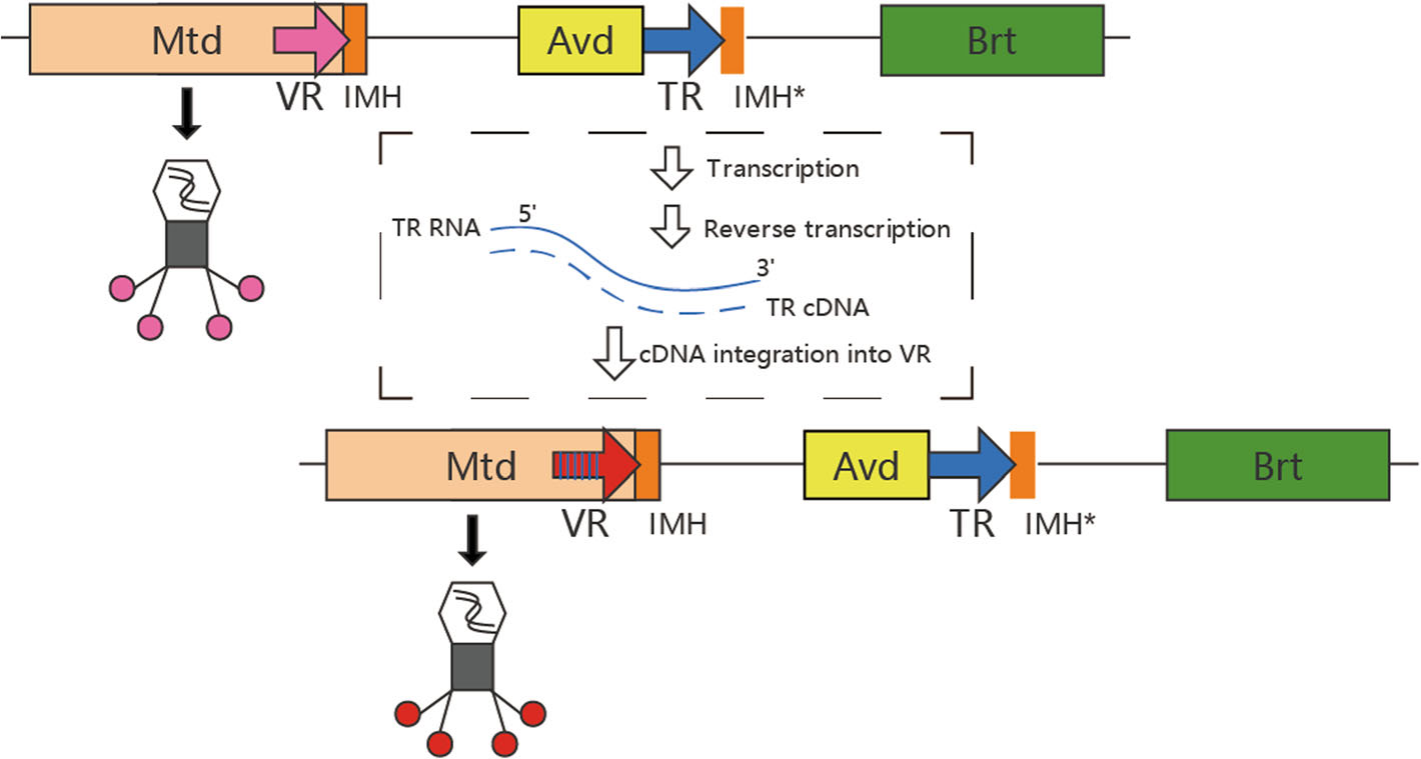
DGR in Bordetella phage BPP-1. Bordetella phage DGR is mainly composed of a target gene (Mtd) with a variable repeat (VR) in the tail, a template repeat (TR), a reverse transcriptase gene Brt (RT) and an accessory gene (Avd)

BPP-1 DGR is composed of a reverse transcriptase gene Brt (RT), a template repeat (TR), a variable repeat (VR) at the end of Mtd (the target gene), and an accessory gene (Avd) to aid tropism switching (Fig. 1). These elements are located adjacently and their functions are closely related. Reverse transcription mediated by Brt gene is the key procedure of diversity-generating mechanism, in which adenine-specific mutagenesis (A-to-N substitution) occurs and TR cDNA is generated. TR cDNA is then integrated into the homologous VR region (cDNA integration), which may diverse the target gene. Other elements also involve in the diversity-generating process: IMH sequence is the initiation of mutagenic homing at the end of VR, IMH* has a similar copy at the end of TR, and Avd (Accessory variability determinant) acts as an accessory gene interacts with RT [10], which is essential for the cDNA synthesis [9].

In the first a couple of genomic surveys, only 155 DGRs were detected in more than 6,000 prokaryotic and phage genomes, indicating that DGRs exist but are rare in the bacterial genomes [11, 12]. Many more DGRs were found using metagenomic datasets. For example, 271 non-redundant DGRs were found in human microbiomes and a new lineage with 1,136 unique DGRs were discovered in the groundwater metagenomes [5]. These indicated the importance to study DGRs using metagenomic methods and datasets. To date, there are thousands of metagenomes sequenced, and a fast analysis tool is in a great demand for the systematic study of DGRs.

Several bioinformatics software or tools have been designed to identify DGRs, such as DiGReF [11], DGRscan [13] and the pipeline applied in the study of groundwater metagenomes [5], all of which were based on sequence alignment. However, these alignment-based approaches are time-consuming with the exploding increase of referential DGRs as well as the sizes of metagenomes. Besides, some larger DGRs may be ignored due to the limited search space or the speed of the program. Another limitation of alignment-based methods (like BLAST) is that they may not work if VR and TR are diverse enough.

TR, VR and RT are necessary elements in DGRs, and they were reported to contain some remarkable motifs [11, 14]. For example, “AAC” tandem repeat is a noteworthy signal in TR motifs (Additional file 1: Fig. S1), which will cause the variation of the target gene if any of the two adenines is replaced by other bases (A-to-N substitution). In this study, we tried to identify sequence patterns of these elements, which were subsequently used in the prediction of candidate TRs, VRs and RTs. Based on these patterns, we developed a computational software, Metagenomic Complex Sequence Scanning Tool (MetaCSST), to identify DGRs. Sequence alignment is no longer required, and the efficiency of our approach is independent of the DGR reference size. The application of MetaCSST to bacterial genomes and human metagenomes generated more DGRs. What’s more, we also discovered several new features of the evolution, variations and functions of DGRs, which may improve our understanding about DGRs.

## Results

We developed MetaCSST system and evaluated it on the referential DGRs as well as those discovered in the human gut virome data (see Methods). We then applied MetaCSST to bacterial genomes and human metagenomes, which together formed 948 unique intact DGRs. Compared with 610 unique known DGRs in bacterial genomes and human metagenomes (see Methods), our finding expanded the number of known DGRs by about 55%.

### Development and evaluation of MetaCSST

In general, MetaCSST was built based on sequence patterns and Generalized Hidden Markov Model (GHMM) (see Methods). TR, and RT were reported to contain remarkable motifs [14], such as “AAC” tandem repeat in TRs (Additional file 1: Figure S1). As the pipeline displayed in Additional file 2: Figure S2, we extracted the most significant sequence patterns of these elements using motif searching tools, and then built several Position Weight Matrices (PWMs) for each element. These PWMs were regarded as states of GHMM. From the referential DGRs, we got the distribution of relative location (states transition matrix) and distance between these states (length distribution), which together with PWMs formed GHMM. Candidate TRs, and RTs were subsequently identified using corresponding GHMMs. Besides, VRs were searched based on the identified TR sequences, and we filtered the predictions to reduce false positive discoveries. This model was able to learn the characteristics of known DGRs and find novel DGRs with similar features. When searching DGRs with MetaCSST, we no longer need to run sequence alignment against a large number of referential DGRs. Therefore, this approach is more efficient than alignment-based tools, especially when a large-scale reference datasets were used.

### Evaluation of MetaCSST

On the referential DGR set (see Methods), 10-fold cross validation showed that our model had the sensitivity and precision of 74.2 and 87.8%, respectively (Additional file 6: Table S1). In addition, we tested our model in 29 DGRs discovered in the human gut virome data [15], and most of these DGRs (25 out of 29; 86.2%) were successfully identified. It’s also worth mentioning that 16 TR-VR pairs from 10 DGRs in this dataset omitted by DGRscan were discovered by MetaCSST (Additional file 7: Table S2). For example, we detected a new TR-VR pair in the sequence of gi|377806301|gb|JQ680373.1|, which shared 85% nucleotide identity and generated 17 A-to-N substitutions.

Except the intact DGRs with all three elements (TR, VR and RT), some partial DGRs can also be discovered because MetaCSST is able to identify these elements separately. Considering the low sensitivity and high false positive rate of predicting VRs independently, we only considered partial DGRs with at least one TR and RT. Given that the metagenomic assemblies are not perfect, the identification of partial DGRs can improve our understanding of DGRs in natural environments.

We applied MetaCSST and DGRscan to HMASM dataset (see Methods) and compared the results from MetaCSST with those from DGRscan. In total, MetaCSST successfully identified 825 intact DGRs and 1,353 partial DGRs (with redundancy, Additional file 15: Data 1c), covering most of DGRs (690 of 837; 82.4%) identified by DGRscan. For the partial DGRs, we aligned the whole genome sequencing (WGS) raw reads to the corresponding VRs (see Methods), and we found 3,036 TR-VR pairs with at least two supporting reads, which resulted in 62.7% of the partial DGRs (848 of 1,353) confirmed (Additional file 15: Data 1c). We collected RTs in the intact DGRs and confirmed partial DGRs, and then removed the redundancy using cd-hit with a threshold of 90% nucleotide identity. In total, we obtained 361 non-redundant RTs, which were over 30% increase compared to the 271 unique DGRs identified by DGRscan.

We also compared the efficiency of MetaCSST and DGRscan for handling HMASM dataset with different sizes of RT reference databases (Additional file 3: Figure S3). It turned out that MetaCSST was about two to three times faster than DGRscan in general, and MetaCSST was faster even when only 155 reference RTs were used for DGRscan.

In summary, MetaCSST could identify most of known DGRs, and capture several novel DGRs missed by DGRscan. What’s more, MetaCSST also identified quite a number of partial DGRs, which were supported by WGS data. This provided a lot of insights for the study of DGRs with metagenomic datasets.

### DGRs in human microbiomes

We applied MetaCSST to the HM dataset (see Methods; Additional file 8: Table S3) and then built a non-redundant DGR reference for human microbiomes with the nucleotide identity of 90% for RTs. Based on this large scale metagenomic study, we constructed a comprehensive set of DGRs in human metagenomes. With intact DGRs and confirmed partial DGRs considered, we detected 656 non-redundant DGRs in total (Additional file 15: Data 1b-1 g; Additional file 16: Data 2), which formed a relatively complete reference set for the subsequent studies of DGRs in the natural environment. The raw sequencing reads were mapped to these DGRs using Bowtie2 [16] and we calculated the coverage depth for each DGR (see Methods). In total, 549 DGRs (83.7%) came from samples with WGS data and all of them were supported by reads, with the average coverage depth of 60.6X (Additional file 9: Table S4; Additional file 4: Figure S4).

### Preference of DGRs in different human body sites

**Fig. 2:**
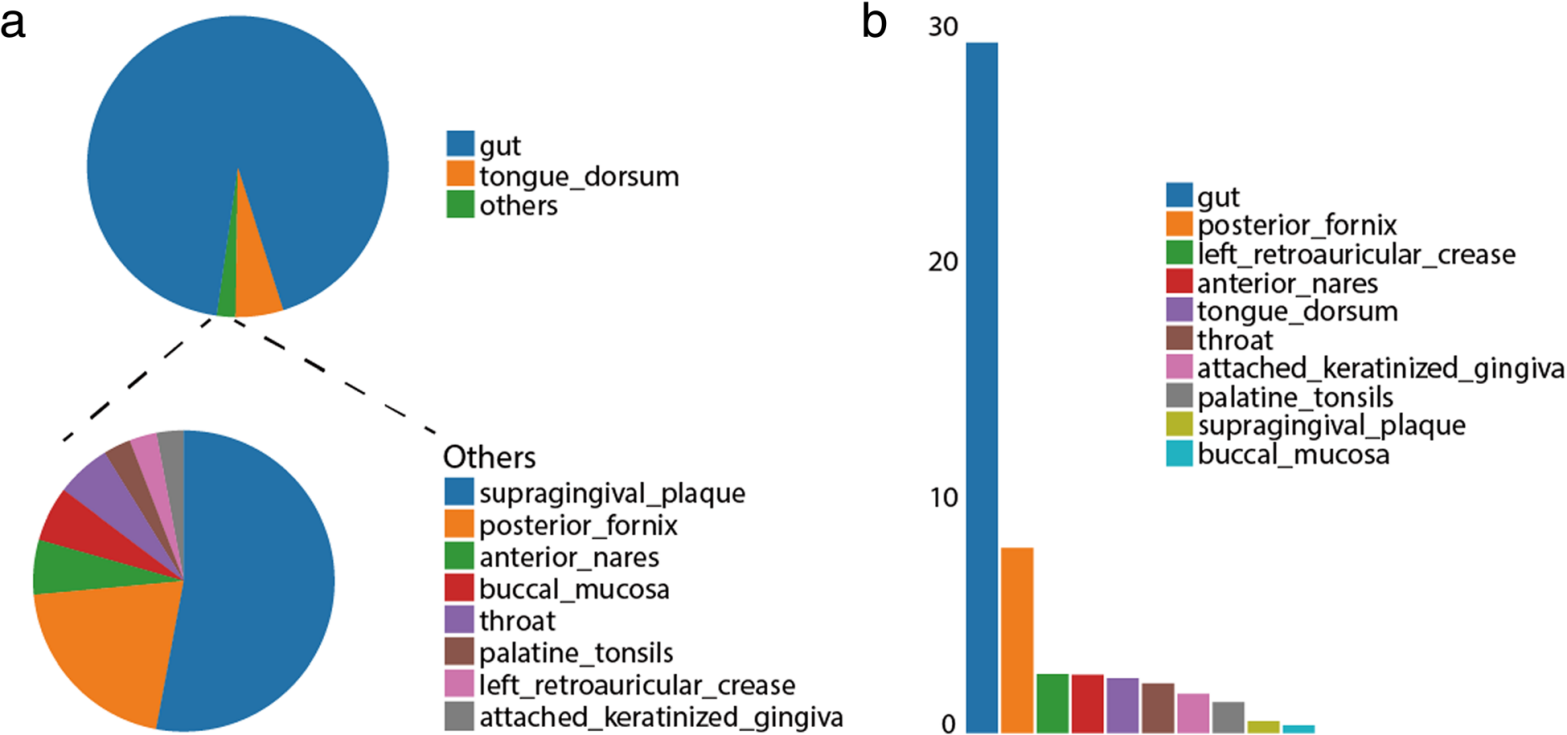
Distribution of DGRs in different body sites. (**a**) Absolute numbers of DGRs in the 10 body sites, by which the legend is sorted. (**b**) The normalized frequencies of DGRs in these body sites: DGR_number_per_100Mbp_contigs = number of DGRs found in this body site * 100 / assembly size (Mbp)

The HMASM dataset contained 749 metagenome samples from 16 human body sites, in which the distribution of DGRs showed considerable biases. We found 1,674 DGRs (intact DGRs and confirmed partial DGRs, with redundancy) in total in the HMASM dataset (Additional file 15: Data 1c), covering 228 samples (30.4%) and 10 body sites. DGRs were much more likely to be found in gut than any other body sites (Fig. 2a), and the normalized frequency also showed uneven distribution, biased to gut and posterior fornix (Fig. 2b).

We got 361 non-redundant DGRs from HMASM dataset with the nucleotide identity of RT < 90%, most of which (92.2%; 333 of 361) were from gut samples. We then ran multiple sequence alignment of RTs of these unique DGRs using mafft-7.310 [17] with default parameters and built a phylogenetic tree using FastTree [18]. The tree was then visualized and annotated with iTOL [19]. The evolution of DGRs was found to be associated with the body sites (Additional file 5: Figure S5). For example, some DGRs from tongue dorsum were clustered in a small branch, indicating that new lineages may exist in different body sites. However, only 28 DGRs came from body sites other than gut, and the result would be more evident if more DGRs from these body sites could be used to build a phylogenetic tree.

In a previous study, DGRs from groundwater metagenomes were found to form novel lineages which were not closely related to the known DGRs discovered in bacteria and archaea [5]. It indicated that there might be some poorly studied lineages of DGRs in the other body sites and they were less likely to be discovered by MetaCSST, a motif-based method.

### DGRs in bacteria with complete genomes

To figure out the distribution of DGRs in bacteria with complete genomes, we applied MetaCSST to BCG dataset (see Methods). We identified 500 unique DGRs (Additional file 15: Data 1a) with the nucleotide identity of RT < 90%. Most of DGRs (95.8%; 479 of 500) were in Proteobacteria, Firmicutes, Bacteroidetes, Actinobacteria and Cyanobacteria, consistent with the result in a recent genomic survey [14]. The classification in different levels can be seen in Additional file 10: Table S5. For instance, DGRs were more likely to be found in Pseudomonas, Bacteroides and Burkholderia at the genus level.

### DGR evolution with diverse cassette patterns

Overall, combining the HM and BCG datasets, we identified 948 non-redundant DGRs (Additional file 17: Data 3). It expanded greatly the scale of DGRs in bacteria genomes and human microbiomes, and thus it could be used as a more comprehensive reference in the following studies.

These 948 non-redundant DGRs are divided into 122 groups of DGR cassette patterns (Additional file 18: Data 4), according to the order, orientation and the frequency of the three substructures (TR, VR and RT). Over half of DGRs belong to the prototypical structure in BPP-1 DGR (G1), while the remaining DGRs are with different cassette patterns. In these diverse cassette patterns, location change (G3), inversion of orientation (G5), and multiple VRs (G2, G4) (compared with G1 cassette) are some of the next most abundant patterns. Statistical result shows that the frequencies of DGRs with different cassette patterns differ greatly. For example, RT is inversed in G5 cassette, compared to the canonical G1 cassette, and the frequency of corresponding DGRs for G1 is more than 20 times higher than that of G5. The bias to some specific DGR cassettes indicates that the order, orientation and the frequency of these substructures may play an important role in the functional process.

**Fig. 3:**
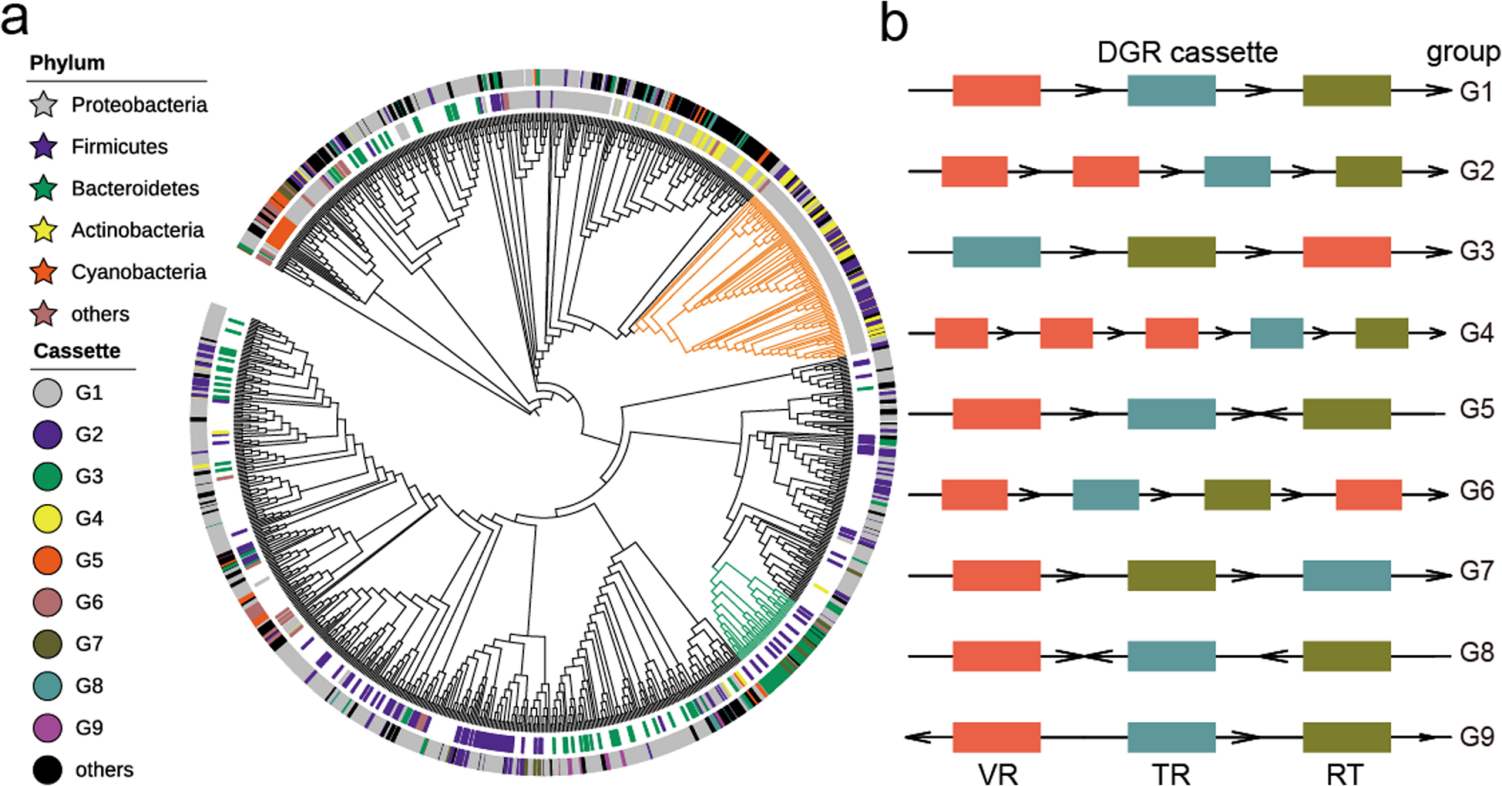
(**a**) Phylogenetic tree with phylum classifications and cassette patterns marked. The inner ring is for the labels of phylum classifications, marked with six colors. If DGRs are from metagenomic datasets, their phylum classification labels will not be marked. The outer ring is for the labels of DGR cassette pattern groups. Except for the top nine cassette patterns, the other 113 cassettes are all marked in black. The green branches highlighted in the phylogenetic tree contains most DGRs with G3 cassette, while most of the orange branches are for DGRs with G2 cassette and G4 cassette. (**b**) Top nine cassette patterns, ranked by corresponding DGR numbers. TR, VR and RT are represented by rectangles filled with different colors; the arrows represent the directions of elements in the DNA sequence, with a rightward arrow representing positive strand and a leftward arrow representing complementary strand

To figure out whether DGR cassette pattern is related with DGR evolution, we constructed the phylogenetic tree (Fig. 3a) using RT genes. Surprisingly, DGRs with different cassette patterns are clearly clustered in some distinct branches. Most notably, the location of VR changes in G3 cassette and DGRs with this structure are clustered in a small branch, highlighted in green. Meanwhile, G2 and G4 cassettes contain multiple VRs and they are different only in the number of VRs, and DGRs with these two structures huddle together in another clade, highlighted in orange. Besides, DGRs in different phylum form different lineages, and DGRs from human microbiomes are clearly separated from those discovered in sequenced genomes. We conducted Chi-square test (see Methods) and it showed that DGR cassette patterns are significantly related to the phylum classification of their source organisms (*p*-value < 2.2e-16) (Additional file 11: Table S6). For instance, DGRs with G2 cassette and G4 cassette are more likely to be found in Proteobacteria (*p*-value = 0.00234 and 2.971e-05, respectively), while DGRs with G3 cassette prefer to come from Firmicutes (p-value = 0.00277).

In summary, the DGR cassette pattern classification is closely related with their source organisms and DGR evolution. The distinctions between DGRs from sequenced genomes and human microbiomes, and the evolutionary distance between DGRs in different phylum indicate that DGRs in different living environments formed new lineages.

### Frequency of non-A-to-N substitutions

**Fig. 4:**
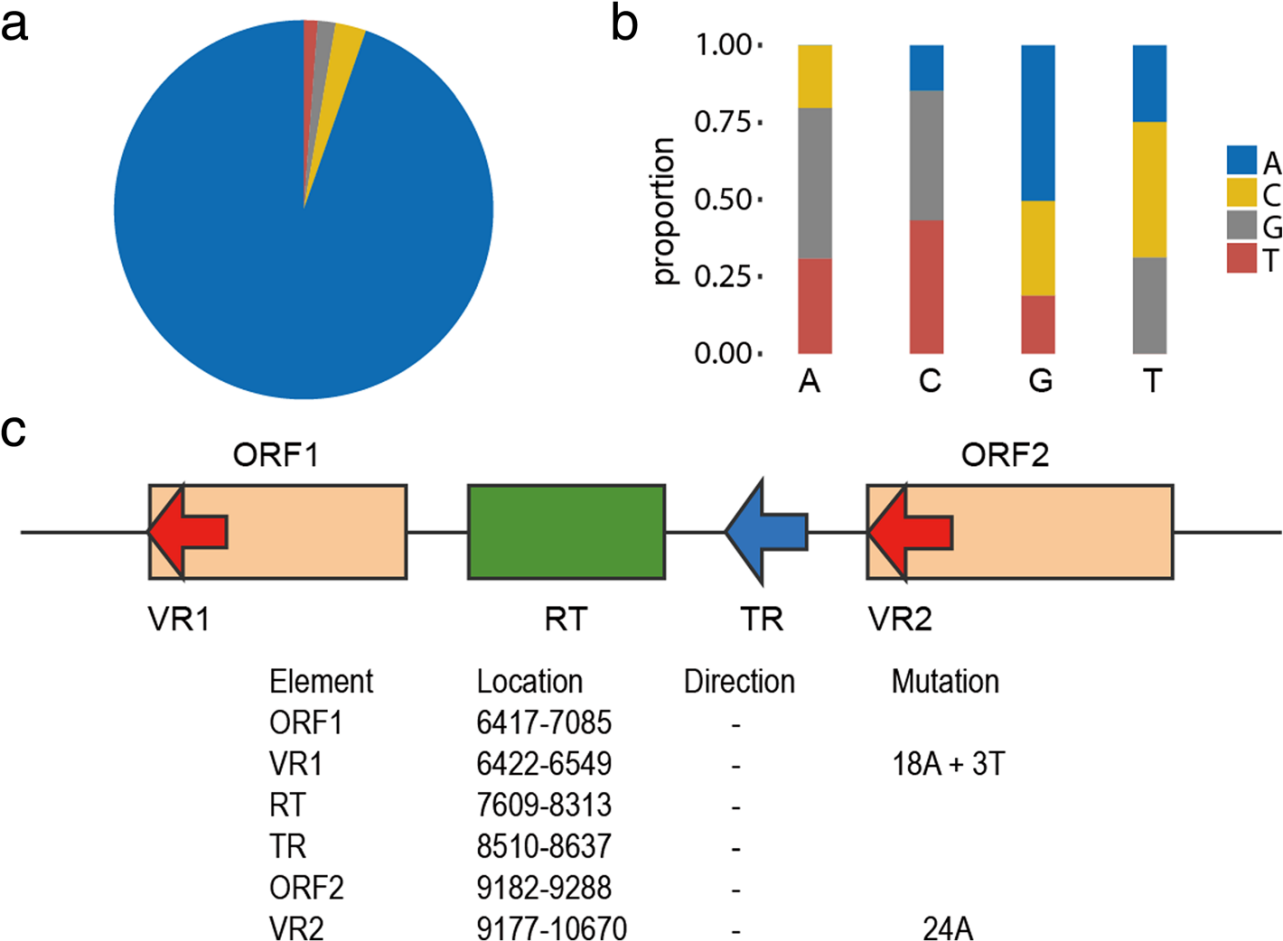
Statistical results of base mutations in non-redundant DGRs (**a**, **b**) and an example of DGR with Non-A-to-N substitutions (**c**). (**a**) Proportions of mutated bases in TRs; (**b**) Directions of base mutations. The four mutated bases are shown in different columns, and the mutation direction is shown by a ratio; (**c**) This DGR from NZ_JIDJ01000049.1 contains one TR template and two VR elements, which located in C-terminal of target genes. In VR1, three thymine were substituted by other bases

The first DGR was discovered in Bordetella Bacteriophage BPP-1, in which the base mutation was described to be adenine-specific [1]. However, the mechanism of adenine-specific mutation is poorly studied in the previous studies. Here we summarized the mutation patterns from 948 non-redundant DGRs and the result showed considerable biases. Overall, the substitution of adenine (A-to-N substitution) dominates the diversity-generating process, while the substitution of other three bases (Non-A-to-N substitution) is also possible (Fig. 4a). Non-A-to-N substitution occurs in 46.3% of TR-VR pairs (868 of 1,874), covering 34.0% non-redundant DGRs (332 of 948). There are 98 TR-VR pairs that contain only Non-A-to-N substitutions, covering 26 unique DGRs. For each TR-VR pair, there is 15 A-to-N substitutions in average, while only about 1.5 Non-A-to-N substitutions. In addition, about half of replaced adenines turn into guanines in the corresponding VRs, and cytosine is the least likely to become adenine, while guanine is more likely to become adenine and thymine prefers to be replaced by cytosine (Fig. 4b).

In BPP-1 DGR, Non-A-to-N substitutions were detected, but at an extremely low frequency (0–0.5%) [20]. However, the frequency of Non-A-to-N substitution (number of Non-A-to-N substitutions / total base number) is 2.58% in average for these 868 TR-VR pairs, while at 2.68% for 98 TR-VR pairs that contain only Non-A-to-N substitutions.

In order to compare, we also summarized 850 VRs identified in HMP dataset by DGRscan program. Non-A-to-N substitutions are found in 44.6% VRs (379 of 850), and the average frequency is 1.47%. From the above, the frequency of Non-A-to-N substitutions might be undervalued in previous studies.

A representative DGR with Non-A-to-N substitutions comes from NZ_JIDJ01000049.1 (*Vibrio cholerae strain 1311–69*), which contains one TR template and two VRs (Fig. 4c). In VR1, three thymine are substituted and the frequency of Non-A-to-N substitutions is 2.36%. These VRs are located in C-terminal of distinct Open Reading Frames (ORFs), which may serve as target genes. Using protein-BLAST tool against nr database (non-redundant protein sequences, updated on 2019/02/28), ORF1 and ORF2 are both DUF1566 domain-containing proteins, which were reported to be the most common PFAM domain contained by target genes of DGRs [5, 13]. Moreover, DUF1566 domains were recently reported to have CLec-folds [21], and putative pilin structures, lipoproteins, fimbrial protein FimH as well as a rearrangement hotspot (rhs) toxin were identified in DUF1566 domains linked to putative transmembrane proteins [5].

### Identification of DGRs with plenty of substitutions

Alignment-based tools like BLAST use “seed” (short identical sequence fragments between TR and VR) to detect homologous sequences. However, huge variation exists in some VR copies compared with their corresponding template repeats, and they will be left out by BLAST if the continuous identical base sequence between TR and VR (CCS; continuous consistent segment) is shorter than the seed length. Here, candidate TRs were identified by MetaCSST independently, and several novel VRs with a number of substitutions were discovered. In the 948 non-redundant DGRs, we discovered 464 TR-VR pairs from 138 DGRs (Additional file 12: Table S7), with the longest CCS shorter than 11 (default seed length of BLASTN). For example, DGR from NZ_FVTJ01000021.1 *(Mycobacteroides abscessus subsp. abscessus strain 490*) contains a variable repeat up to 125 bp, in which 34 adenines are replaced and the longest CCS is only 9 bp, and this TR-VR pair will be left out by BLASTN with default parameters.

### Multiple VRs and their impact on target genes

The “copy and paste” process is the key procedure in the diversity-generating mechanism. In this process, DGRs with multiple VRs could be generated when the reverse transcript cDNA segments from an original TR region were inserted into many positions or DGRs contain multiple original TRs and corresponding VRs, leading to a remarkable mutation of the genome. About 28.6% non-redundant DGRs (271 of 948) contain multiple VRs, most of which (79.9%; 216 of 271) include only one template repeat, indicating that a single TR can generate multiple VRs. Surprisingly, a single TR in some DGRs can generate a dozen or even hundreds of VR copies, which can be called a highly repetitive TR. For example, DGR from NZ_FNTS01000002.1 (*Pseudomonas costantinii strain BS2773*) contains 6 unique TRs and 91 TR-VR pairs, which is an atypical DGR with Non-A-to-N substitutions but few A-to-N substitutions. In average, the identity between TR and VR is 97.6% and the VR length is about 122 bp. Such highly repetitive TRs exist in DGRs from some organisms, such as NZ_JXDL01000001.1 (*Bradyrhizobium sp. AT1 scaffold00001*), NZ_KE384013.1 (*Mesonia mobilis DSM 19841 G551DRAFT_scaffold00002.2*), etc.

To figure out the location of VRs in the target genes, we searched Open Reading Frames (ORFs) in the DGR containing sequences and summarized the location relationship between VRs and the ORFs of their target genes (Additional file 13: Table S8). Generally, most of DGRs (86.7%; 235 of 271) with multiple VRs involve multiple target genes, while there are 31 DGRs (11.3%) contain multiple VRs which overlap with a same target ORF.

**Fig. 5:**
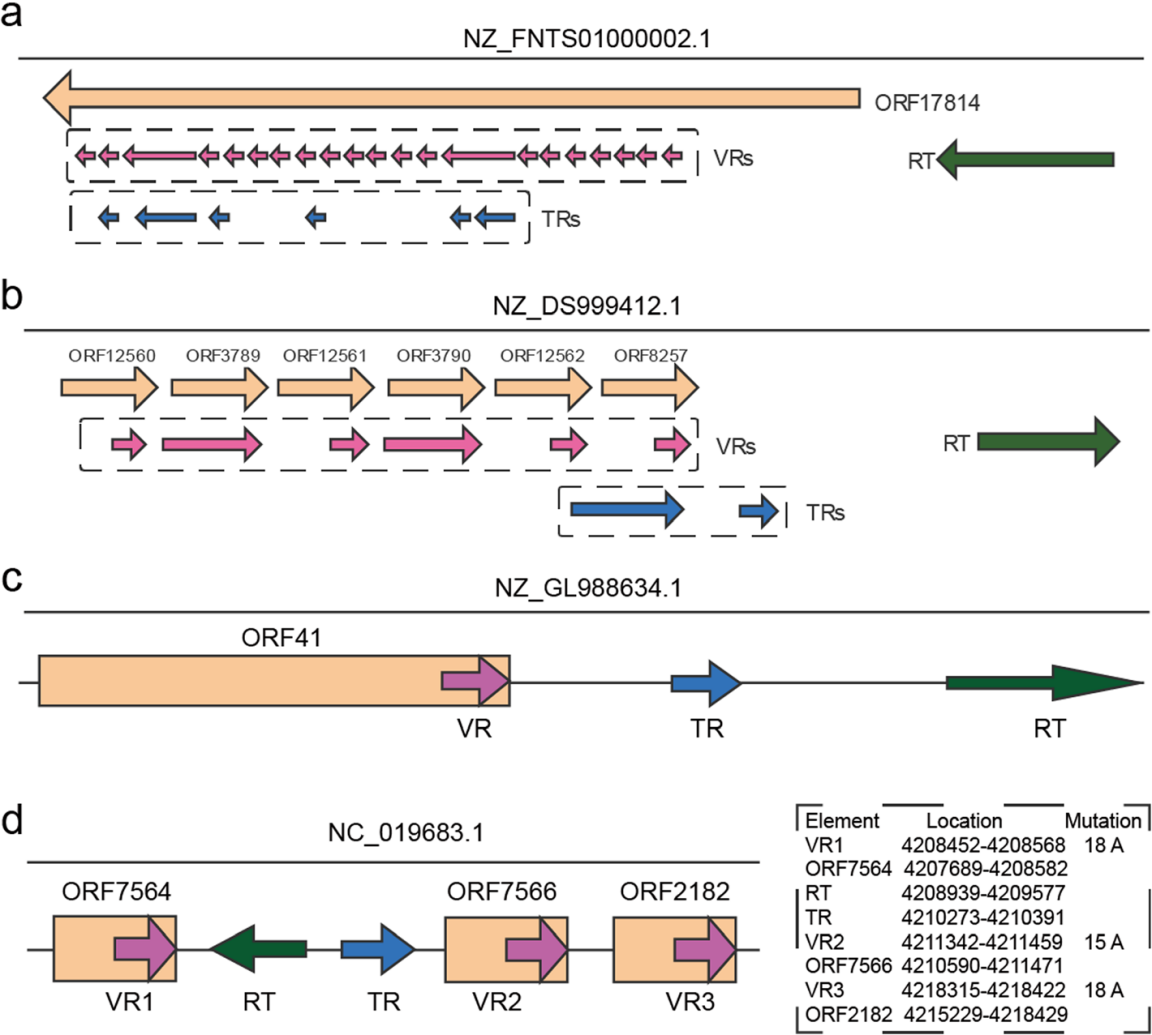
DGR examples. TRs, VRs, RTs and target genes (ORFs) are filled with different colors, while the arrow direction represents the direction of these elements. (**a**) DGR found in NZ_FNTS01000002.1 contains 21 VRs within ORF17814, thus introducing huge variation to this target gene; (**b**) DGR from NZ_DS999412.1 contains six VRs, involving different target genes; (**c**) NZ_GL988634.1 has a typical DGR structure similar with BPP-1, and the target gene (ORF41) serves as phage tail domain protein; (**d**) An untypical DGR from NC_019683.1. It contains three VRs that are all located in C-terminal of target genes

VRs from several DGRs are adjacently localized in the same target gene, thus leading to a remarkable variation of this protein. For instance, DGR from NZ_FNTS01000002.1 (*Pseudomonas costantinii strain BS2773*) contains 21 merged VRs (some VRs may be overlapped with each other, and they are merged based on genomic coordinates), and they are all located within NZ_FNTS01000002.1|ORF17814 (Fig. 5a). We searched this protein in NR database using BLASTP (https://blast.ncbi.nlm.nih.gov/Blast.cgi). The top three hits (ranked by identity) are WP_074851656.1 (large adhesive protein [*Pseudomonas costantinii]*), WP_100831476.1 (LapA family giant adhesin [*Pseudomonas tolaasii*]) and OPA96034.1 (large adhesive protein [*Pseudomonas fluorescens*]). Among this, WP_074851656.1 shares 100% amino acid identity and 4623aa with NZ_FNTS01000002.1|ORF17814. It appears that this gene functions as adhesin, which enable bacteria to adhere to host cells. The interaction between adhesins and their complementary receptors on host cell surfaces determines the bacterial attachment to host tissue surfaces [22]. To some extent, this target gene has similar functions as Mtd in BPP-1 DGR, to bind the specific receptor on host surfaces. In the meanwhile, in some DGRs, different copies of VRs overlap with various ORFs, thus leading to the diversification of multiple target genes. DGR from NZ_DS999412.1 (*Stenotrophomonas sp. SKA14 scf_1108481805244*) is a such kind of DGR, which contains two TRs and six merged VRs in different ORFs (Fig. 5b). Similarly, we searched homologous proteins in nr database for these ORFs. It turned out that five of them belonged to DUF1566 domain-containing proteins, while the left one remained unknown.

### Target genes and putative functions

For the 948 non-redundant DGRs, we searched the ORFs (see Methods) in the DGR containing sequences, and it turned out that most of VRs (92.6%; 1,509 of 1,629) overlapped with the ORFs (or contained in the ORFs). The wide distribution of VRs in putative protein coding segments revealed the existence of influenced target genes by DGRs. We got the longest overlapped ORF for each VR, which was regarded as the putative target gene of the DGR. For these target genes, we ran BLASTP to nr database (see Methods) and selected the best match with the maximum identity*length to represent the target gene. It turned out that 1,114 genes (77.6%) had matches in nr database (Additional file 14: Table S9). Among this, 723 genes (64.9%) are labeled with “hypothetical protein”, while 63 genes (5.7%) are “uncharacterized protein”. The next abundant annotation is DUF1566 domain-containing protein (41 genes, 3.7%), the most common PFAM domain contained by target genes of DGRs [5, 13].

What’s more, two target genes are adhesive proteins (NZ_FNTS01000002.1|ORF17814 and NZ_JXDL01000001.1|ORF29788), which enable bacteria to adhere to host cells through binding the specific receptor molecule on host cell surfaces [22]. NZ_GL988634.1|ORF41 is matched to EGS66981.1 (100% identity with 614aa), which serves as phage tail domain protein. This DGR has a typical cassette structure (G1), in which TR and VR share 90.2% identity with 12 A-to-N substitutions (Fig. 5c). Besides, several functions are correlated with membrane structure, such as “membrane protein”, “SusC/RagA family TonB-linked outer membrane protein”, “sugar ABC transporter integral membrane protein” and so on. Target genes are also involved in various functions, such as a variety of enzymes, flagellin, transporter, ATP-binding, sulfatase-modifying, etc. For instance, in DGR of NC_019683.1, a TR generates three VRs with A-specific mutagenesis, which are all located in C-terminal of corresponding target genes (Fig. 5d). These target genes are matched to WP_015135700.1, WP_015135693.1, and WP_015135696.1 respectively (100% identity). WP_015135700.1 serves as phytochrome sensor protein, while WP_015135693.1 and WP_015135696.1 are sulfatase-modifying factor proteins in *Leptolyngbya sp. PCC 7376*. It demonstrates that this DGR doesn’t help to bind receptor molecules on host cells, but influence phytochrome sensor and sulfatase-modifying.

In conclusion, several novel target proteins are identified, like adhesin and phage tail domain protein, which function similar with BPP-1 DGR to a large extent. Besides, target genes are also found to be correlated with many other functions, indicating that DGR might be a universal mechanism to generate diversity and have a wide impact on multiple functional processes.

## Discussion

Several remarkable characteristics were used to identify DGRs since they were discovered, such as reverse transcriptase, base mutation in TR-VR pairs, target genes and so on. Base mutation was a significant signal in DGRs, which was regarded to be adenine-specific. For instance, other bases (T, C and G) were not permitted to be replaced in the pipeline of DiGReF [11]. With a slight flexibility, a small fraction of Non-A-to-N substitutions were allowed and a large amount of DGRs were identified [5, 13]. In our study, we further increased the frequency thresholds of Non-A-to-N mutagenesis, and we obtained a large number of DGRs (as Fig. 4c). In summary, Non-A-to-N substitutions should be paid more attention when designing strategies to detect DGRs.

This study identifies DGRs in sequenced bacterial genomes and metagenomes using a non-alignment-based method. Some results of this study are consistent with previous studies, while the other parts are intriguing. Although this study is based entirely on sequencing data analysis, it can be of interest to the retrotransposon element research community. It’s worth noting that Non-A-to-N substitutions implied by this study is different to some recent studies [9, 20], especially, the reconstituted in vitro system based entirely on purified components [23]. Sequencing and assembly errors can be possible explanations to this mutagenesis, though the large numbers of evidences supporting this mutagenesis indicate the possibility might be low. In another way, it is possible that although some of the DGRs exist in the data sets investigated in this study, their activeness is unknown. The previous studies were focused on active DGRs in purified environments, while this study expanded the study to natural environments.

## Conclusions

This large scale genomic and metagenomic study provided a more comprehensive reference dataset of DGRs, and uncovered some new features of DGRs. For DGRs in bacteria genomes and human microbiomes, the non-redundant DGRs we found expanded the number of known DGRs by about 55%. We verified some viewpoints in the previous studies: (1) Sequence motifs exist in the substructures, which can be used to identify new DGRs; (2) Owing to heredity and variation, new lineages of DGRs can be formed in different living environments. (3) Base mutation shows obvious biases, but non-A-to-N substitutions are also possible in TR-VR mutational patterns. In addition, we further illustrated several new characterizations about DGRs: (1) DGR is a powerful mechanism in generating diversity for the ability to cause a huge variation in a single target gene or multiple target genes, and the number of base substitutions can be huge in some VRs; (2) DGRs show preference in different human body sites, indicating that DGRs in different body sites may have distinct lineages; (3) DGR cassette patterns are related to their source genomes, which is especially true for DGRs with G2 and G4 cassette patterns or G3 cassette patterns; (4) Non-A-to-N substitution was underestimated in previous studies; (5) Several novel proteins are proved to be influenced by DGRs, like adhesin and phage tail domain protein, which share similar functions with BPP-1 DGR in the aspect of receptor-binding; (6) The evidence is overwhelming that DGRs have an impact on multiple functional processes in addition to receptor-binding, such as phytochrome sensor, sulfatase-modifying, etc.

## Methods

### Data sets

Assembled results and WGS raw reads of 749 human metagenomic samples (called HMASM dataset) were downloaded from NIH Human Microbiome Project database (HMP; http://www.hmpdacc.org/), and 94 metagenome assemblies of stool samples were collected from HMP as well. In order to study DGRs in uncultured environments with a more comprehensive dataset, we got four other WGS datasets of human gut metagenomes from SRA database (https://www.ncbi.nlm.nih.gov/sra): SRP008047, SRP011011 [24], SRP115494 and ERP019800. In total, 5.17 TB WGS raw data from 3,305 samples were obtained, which was the most comprehensive metagenomic dataset in the study of DGRs up to date. In this paper, the dataset containing these data was called HM (Human Microbiomes) dataset.

In addition to the HM dataset, we downloaded over 81,000 genome assemblies of bacteria from NCBI RefSeq website (ftp://ftp.ncbi.nlm.nih.gov/genomes/refseq/bacteria), which in total were called BCG (Bacterial Complete Genomes) dataset.

#### The referential DGRs

We collected 126 DGRs identified by DiGReF in the complete genomes [11] and 837 DGRs discovered by DGRscan in human microbiomes [13]. These DGRs were considered as referential DGRs and they were used for the model building and evaluation.

#### The known DGRs

DGRs found by the program DiGReF [11], DGRscan [13] and the study of a large-scale genomic survey [14] were collected, which together formed 610 unique known DGRs (with RT nucleotide identity < 90%) in sequenced bacterial genomes and human microbiomes**.**

### Development of MetaCSST

The referential DGRs were randomly divided into ten groups, nine of which were used as training set and the remaining one group as the test set. The training set was used for model building, while the test set was used for evaluation. For DGRs in the training set, TR and RT fragments were extracted according to the referential DGR. Motifs of TRs were identified using GLAM2 with default parameters, while the sequence motif of RT was constructed using MUSCLE v3.8.31 [25] with default parameters. Position Weight Matrices (PWMs) for TRs and RTs were constructed according to the motifs. Each PWMs were considered as a state, and the state-transition matrix and length distribution, counted from the training set using the approach of maximum likelihood, were used to generate a GHMM integrated with PWMs. When searching these substructures, Viterbi algorithm was applied to find out the best prediction with the maximum score.

In order to improve the system performance, the training sets for TR and RT were further clustered and divided into three subgroups, and a GHMM was developed for each subgroup with above pipeline. Therefore, 6 GHMMs were constructed, of which three for TRs and three for RTs (detailed arguments can be found in config files in the MetaCSST package). For VRs, their motifs were not strong enough to identify new VRs separately, they were searched according to corresponding TRs in the genomes or assemblies. VRs were filtered according to the following three criteria: the length is longer than 30 bases, the nucleotide identity is higher than 60%, and the number of Non-A-to-N substitution is under four.

### Evaluation of MetaCSST

Several measurements were used to evaluate our approach: false positive rate (false prediction of TR, VR or RT / sequence number in gold negative dataset), sensitivity (True prediction / all in test set) and precision (True prediction / all predictions by MetaCSST). During this process, we generated four gold negative datasets composed of 100,000 random sequences with the background base frequencies of DGRs, the length of which are 200 bp, 2kbp, 10kbp and 100kbp separately.

### De novo assembly

In total, we collected 2,462 WGS sequencing data of gut samples from SRA database. De novo assembly for each sample was performed by SOAPdenovo2 with the parameters: “-K 39 -R”. In the assembly of samples from ERP019800, the arguments in the config file were set as “251, 500, 0, 3, 251, 1, 3, 32”. As for samples from SRP008047, SRP011011 and SRP115494, the parameters were “100, 200, 0, 3, 100, 1, 3, 32”. Scaffolds longer than 500 bp were integrated with 94 gut assemblies from HMP database and used in the downstream DGR prediction.

### DGR prediction and validation

DGR prediction was conducted using MetaCSST. Partial DGRs were validated with WGS data using NCBI-BLAST-2.5.0+ [26] (“-task blastn, -evalue 1e-5”), to screen out reads that were possible to contain VRs according to candidate TRs. Afterwards, VRs were searched in remaining reads, during which at most three Non-A-to-N substitutions were permitted and they were supported by at least two reads. Afterwards, WGS raw reads were mapped to DGRs using Bowtie2 [16] with default parameters, and the coverage depth for each DGR was calculated using the following formula:

$$ \operatorname{cov}=100\ast \frac{number\kern0.17em of\kern0.17em matched\kern0.17em reads}{\mathrm{se} quence\kern0.17em length}. $$

### Chi-square test

Based on the phylum classification and cassette patterns of the 948 non-redundant DGRs, we built a contingency table. Chi-square test was conducted with R-3.3.2 to test whether the two factors are independent, with the threshold of *p*-value as 0.05. While testing the independence between phylum classification and a specific DGR cassette, all cassette patterns were divided into two groups, for example, G2 group and Non-G2 group.

### ORFs finding

ORFs were found according to six-frame translation, with “ATG” as the only start codon and minimal ORF length of 120 bp (40aa). Each ORF and its protein translation were kept as putative protein encoding segments. This program can be found in the MetaCSST package.

### BLASTP against nr database for target genes

Target proteins were aligned against nr database using BLASTP with the following parameters: “-evalue 1e-5”. Here, the matched proteins were required to share more than 40% amino acid identity, with cover length > 100aa in the meanwhile.

## Additional files

Additional file 1: **Figure S1.** Sequence motifs for the three groups of TRs (DOCX 308 kb)

Additional file 2: **Figure S2.** MetaCSST development pipeline (DOCX 96 kb)

Additional file 3: **Figure S3.** Efficiency comparison with DGRscan (DOCX 28 kb)

Additional file 4: **Figure S4.** Annotated phylogenetic tree of 656 unique DGRs from human microbiomes (DOCX 433 kb)

Additional file 5: **Figure S5.** Phylogenetic tree of non-redundant DGRs from HMP dataset (DOCX 275 kb)

Additional file 6: **Table S1.** Evaluation of MetaCSST. URL: http://cgm.sjtu.edu.cn/index/pub/software/MetaCSST/supplementary/Supplementary_Table_1.xlsx (XLSX 9 kb)

Additional file 7: **Table S2.** DGRs and TR-VR pairs discovered by MetaCSST in human gut virome, which were omitted by DGRscan. URL: http://cgm.sjtu.edu.cn/index/pub/software/MetaCSST/supplementary/Supplementary_Table_2.xlsx (XLSX 11 kb)

Additional file 8: **Table S3.** Data information of human metagenomic datasets. URL: http://cgm.sjtu.edu.cn/index/pub/software/MetaCSST/supplementary/Supplementary_Table_3.xlsx (XLSX 10 kb)

Additional file 9: **Table S4.** Coverage depth for DGRs with WGS data. URL: http://cgm.sjtu.edu.cn/index/pub/software/MetaCSST/supplementary/Supplementary_Table_4.xlsx (XLSX 250 kb)

Additional file 10: **Table S5.** Species taxonomy of DGRs found in bacteria sequenced genomes URL: http://cgm.sjtu.edu.cn/index/pub/software/MetaCSST/supplementary/Supplementary_Table_5.xlsx (XLSX 51 kb)

Additional file 11: **Table S6.** Result of chi-square test. URL: http://cgm.sjtu.edu.cn/index/pub/software/MetaCSST/supplementary/Supplementary_Table_6.xlsx (XLSX 10 kb)

Additional file 12: **Table S7.** DGRs with huge variation and short CCS in TR-VR pairs. URL: http://cgm.sjtu.edu.cn/index/pub/software/MetaCSST/supplementary/Supplementary_Table_7.xlsx (XLSX 50 kb)

Additional file 13: **Table S8.** Overlapped VRs and ORFs. URL: http://cgm.sjtu.edu.cn/index/pub/software/MetaCSST/supplementary/Supplementary_Table_8.xlsx (XLSX 58 kb)

Additional file 14: **Table S9.** Matching result of target genes in nr database. URL: http://cgm.sjtu.edu.cn/index/pub/software/MetaCSST/supplementary/Supplementary_Table_9.xlsx (XLSX 542 kb)

Additional file 15: **Data 1.** DGRs identified in genomic and metagenomic datasets. URL: http://cgm.sjtu.edu.cn/index/pub/software/MetaCSST/supplementary/Supplementary_Data_1.zip (ZIP 3330 kb)

Additional file 16: **Data 2.** 656 unique DGRs from human microbiomes. URL: http://cgm.sjtu.edu.cn/index/pub/software/MetaCSST/supplementary/Supplementary_Data_2.gtf (GTF 881 kb)

Additional file 17: **Data 3.** 948 non-redundant DGRs from genomic and metagenomic datasets. URL: http://cgm.sjtu.edu.cn/index/pub/software/MetaCSST/supplementary/Supplementary_Data_3.gtf (GTF 2105 kb)

Additional file 18: **Data 4.** 122 cassette structures of 948 non-redundant DGRs. URL: http://cgm.sjtu.edu.cn/index/pub/software/MetaCSST/supplementary/Supplementary_Data_4.pdf (PDF 171 kb)

## Data Availability

Assemblies, raw reads of HMASM dataset and 94 metagenome assemblies of stool samples were collected from HMP database (http://www.hmpdacc.org/). We also downloaded four WGS datasets of human gut metagenomes from SRA database: SRP008047, SRP011011, SRP115494 and ERP019800. Besides, over 81,000 genome assemblies of bacteria was obtained from NCBI RefSeq (ftp://ftp.ncbi.nlm.nih.gov/genomes/refseq/bacteria). Known DGRs were collected from previous publications. The MetaCSST package, assemblies, identified DGRs and other materials can be found at http://cgm.sjtu.edu.cn/index/pub/software/MetaCSST/MetaCSST.php. MetaCSST package can also be found in GitHub: https://github.com/fzyan/MetaCSST.

## Abbreviations

CCS: Continuous consistent segment
DGR: Diversity-generation retroelement
IMH: Initiation of mutagenic homing
ORF: Open Reading Frame
RT: Reverse transcriptase gene
TR: Template repeat
VR: Variable repeat
