# Supplementary material for: Discovery and characterization of the evolution, variation and functions of diversity-generating retroelements using thousands of genomes and metagenomes
Source: BMC Genomics. 2019 Jul 19;20:595. doi: 10.1186/s12864-019-5951-3 (PMC6642488; doi:10.1186/s12864-019-5951-3)
Supplement: Supplementary file 1 — Figure S1. Sequence motifs for the three groups of TRs (DOCX 308 kb) [file 12864_2019_5951_MOESM1_ESM.docx]

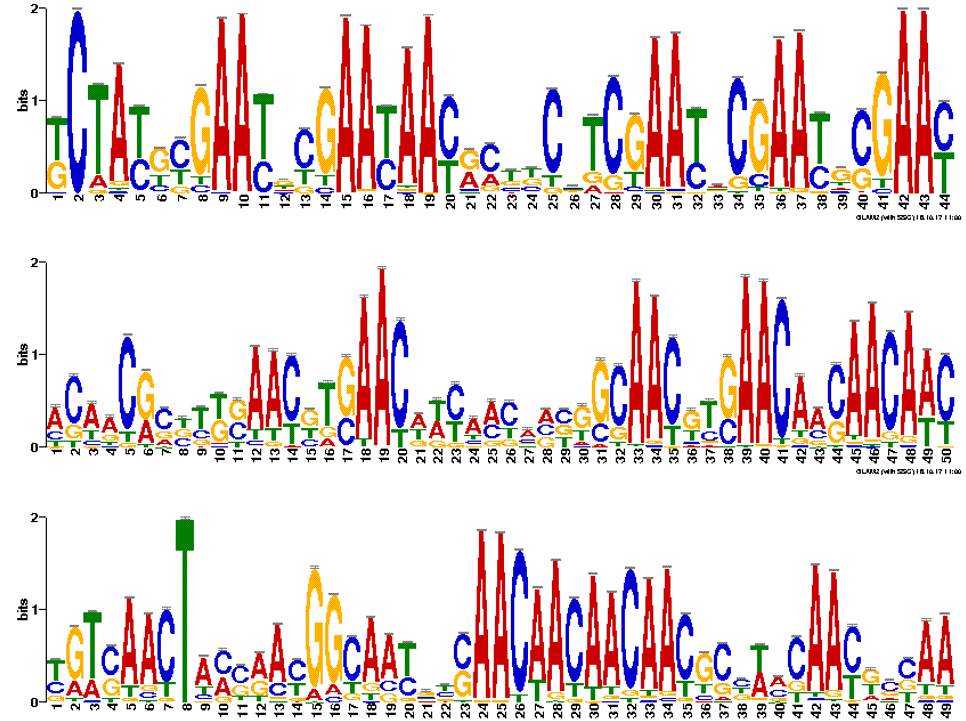


**Figure S1.** TR motifs. TR sequences from the training set are clustered and then divided into three groups. For each group, the sequence motifs are called using GLAM2, and then the motif with the highest score is chosen for visualization here.
