# Supplementary material for: Discovery and characterization of the evolution, variation and functions of diversity-generating retroelements using thousands of genomes and metagenomes
Source: BMC Genomics. 2019 Jul 19;20:595. doi: 10.1186/s12864-019-5951-3 (PMC6642488; doi:10.1186/s12864-019-5951-3)
Supplement: Supplementary file 2 — Figure S2. MetaCSST development pipeline (DOCX 96 kb) [file 12864_2019_5951_MOESM2_ESM.docx]

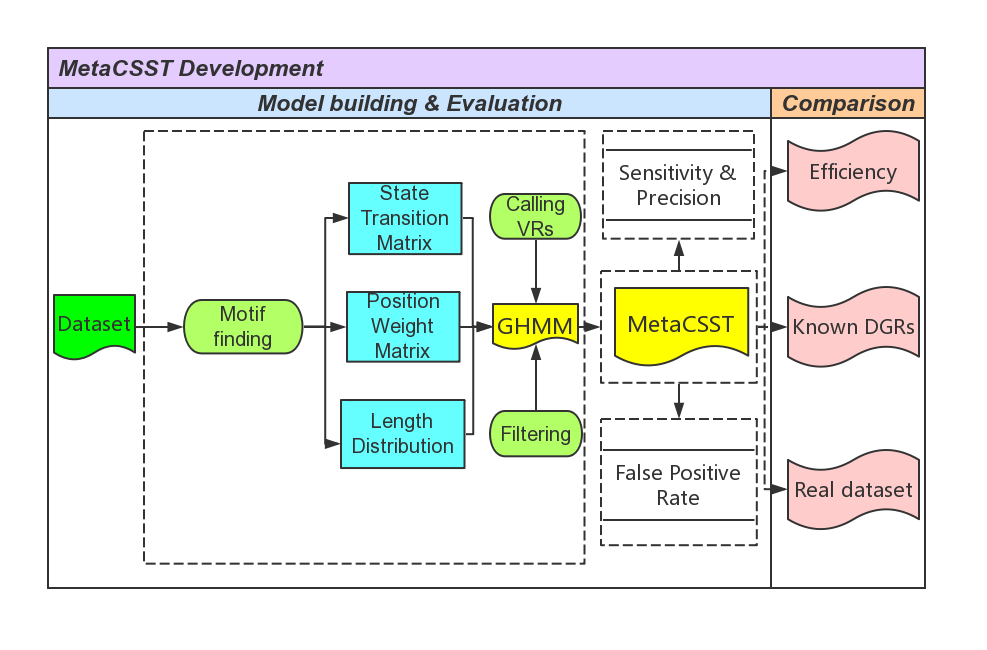


**Figure S2.** MetaCSST analysis pipeline. The dataset we collected were divided into K groups randomly (K=10), and the sequence motifs for TR, VR and RT were called for each group. Afterwards, we built position weight matrices from the aligned matrix, and obtained state transition matrices and length distribution models from the training set in the meanwhile. These models were integrated to generate a generalized hidden Markov model(GHMM), which forms MetaCSST together with the pipeline of calling VRs and DGR filtering. We evaluated the model by the three indexes: sensitivity, precision and false positive rate. Finally, MetaCSST is compared with other software using known DGRs and real dataset as well, evaluated by the efficiency and identified DGRs.
