# Supplementary material for: Discovery and characterization of the evolution, variation and functions of diversity-generating retroelements using thousands of genomes and metagenomes
Source: BMC Genomics. 2019 Jul 19;20:595. doi: 10.1186/s12864-019-5951-3 (PMC6642488; doi:10.1186/s12864-019-5951-3)
Supplement: Supplementary file 3 — Figure S3. Efficiency comparison with DGRscan (DOCX 28 kb) [file 12864_2019_5951_MOESM3_ESM.docx]

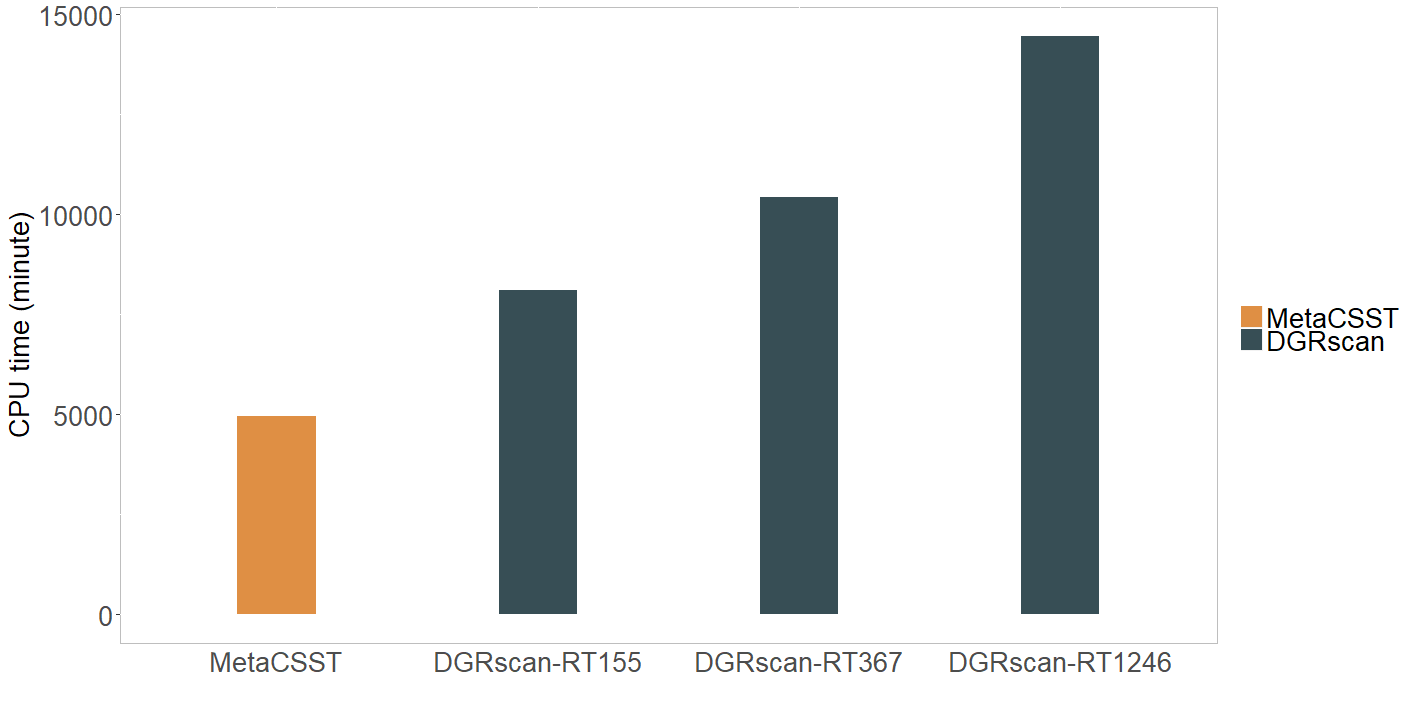


Figure S3. Comparison of efficiency with DGRscan. MetaCSST used 963 DGRs identified by DiGReF and DGRscan, and the CPU time is compared with DGRscan using different RT dataset: (1) RT155: RTs of 155 DGRs identified by DiGReF in sequenced genomes; (2) RT367: 367 unique RTs found by DGRscan in HMP dataset; (3) RT1246: 1,246 non-redundant RTs, including those identified in the groundwater metagenomes.
