# Supplementary material for: Discovery and characterization of the evolution, variation and functions of diversity-generating retroelements using thousands of genomes and metagenomes
Source: BMC Genomics. 2019 Jul 19;20:595. doi: 10.1186/s12864-019-5951-3 (PMC6642488; doi:10.1186/s12864-019-5951-3)
Supplement: Supplementary file 4 — Figure S4. Annotated phylogenetic tree of 656 unique DGRs from human microbiomes (DOCX 433 kb) [file 12864_2019_5951_MOESM4_ESM.docx]

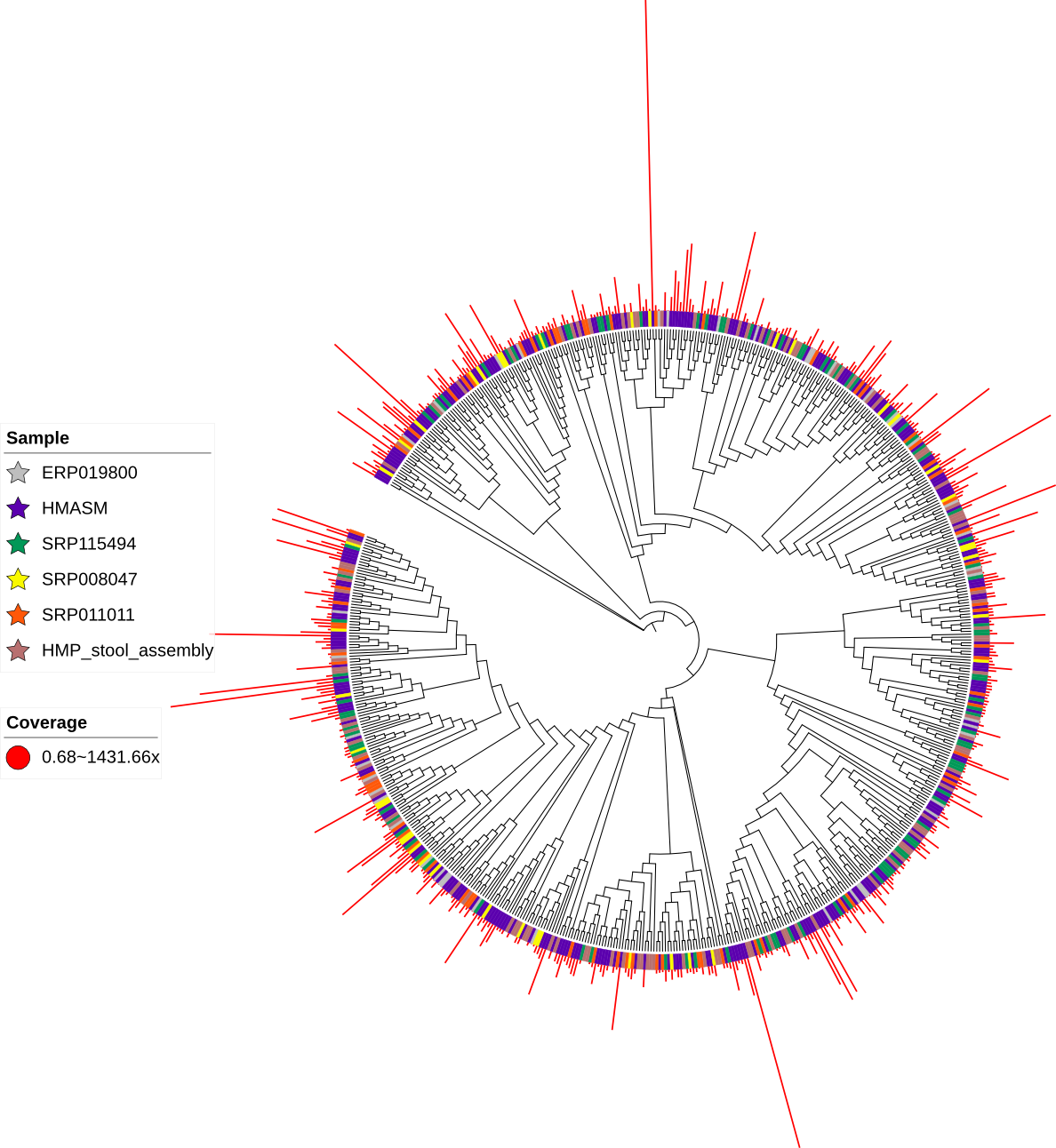


**Figure S4.** Annotated phylogenetic tree of 656 core DGRs from human microbiomes. The inner ring is for the labels of samples, and the outer ring is a bar plot of coverage depths, ranging from 0.68x to 1,431.66x. For 107 DGRs come from HMP stool assembly without WGS sequencing data, their sequencing coverage bar are not shown, and their positions in the inner ring are filled in gray.
