# Supplementary material for: Discovery and characterization of the evolution, variation and functions of diversity-generating retroelements using thousands of genomes and metagenomes
Source: BMC Genomics. 2019 Jul 19;20:595. doi: 10.1186/s12864-019-5951-3 (PMC6642488; doi:10.1186/s12864-019-5951-3)
Supplement: Supplementary file 5 — Figure S5. Phylogenetic tree of non-redundant DGRs from HMP dataset (DOCX 275 kb) [file 12864_2019_5951_MOESM5_ESM.docx]

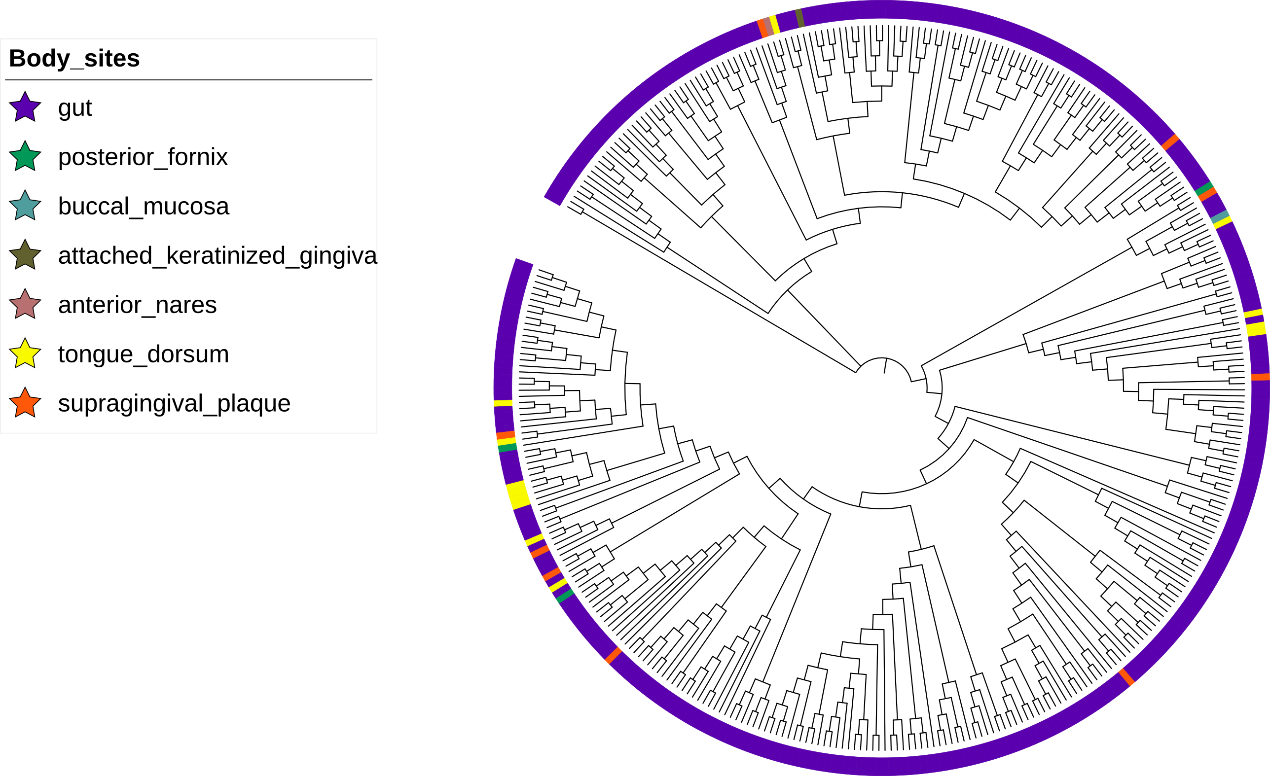


**Figure S5.** Phylogenetic tree of non-redundant DGRs from HMP dataset. These 361 DGRs come from seven body sites, marked with different colors in the strip.
