# Supplementary figures and images for: Discovery and characterization of the evolution, variation and functions of diversity-generating retroelements using thousands of genomes and metagenomes
Source: BMC Genomics. 2019 Jul 19;20:595. doi: 10.1186/s12864-019-5951-3 (PMC6642488; doi:10.1186/s12864-019-5951-3)

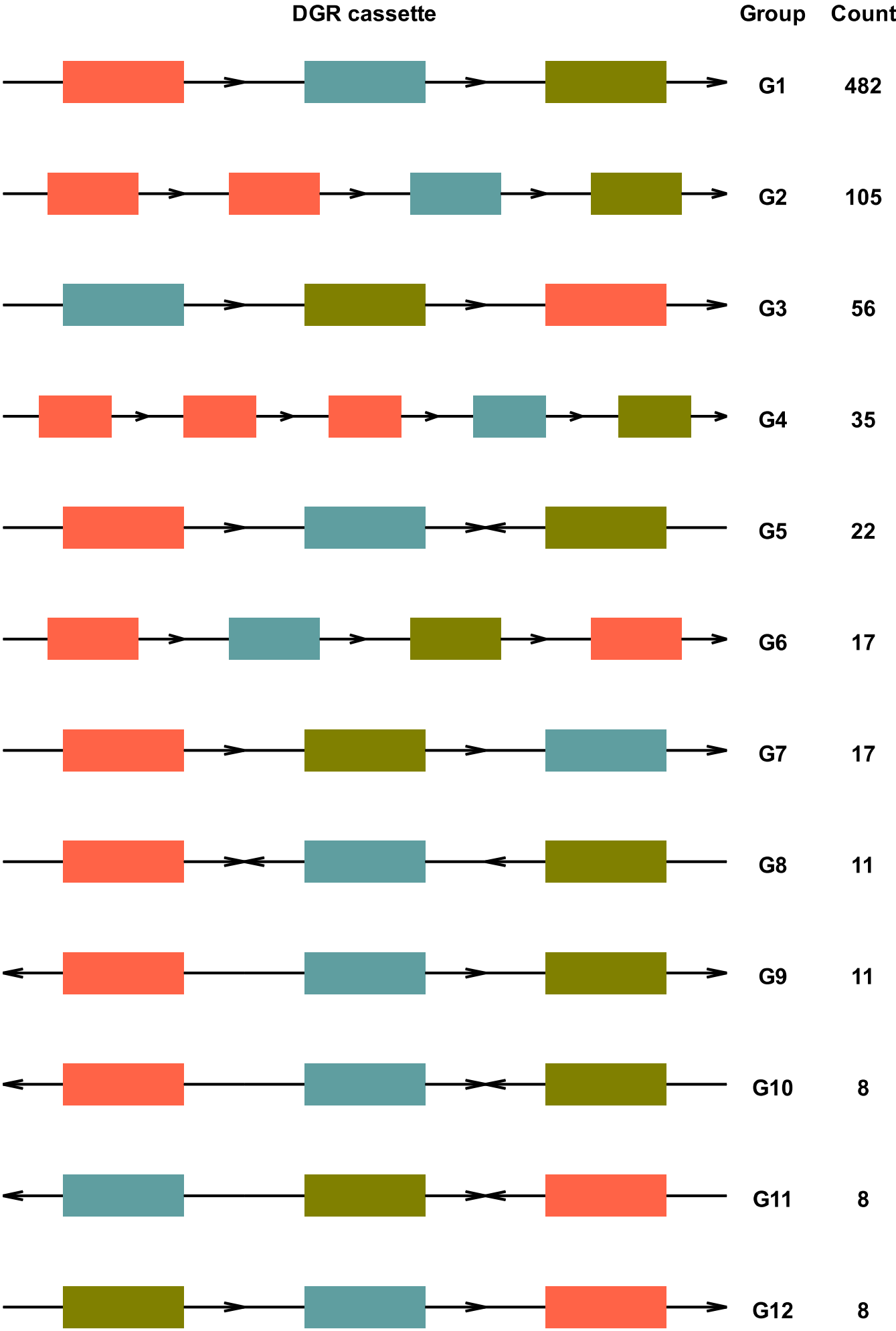

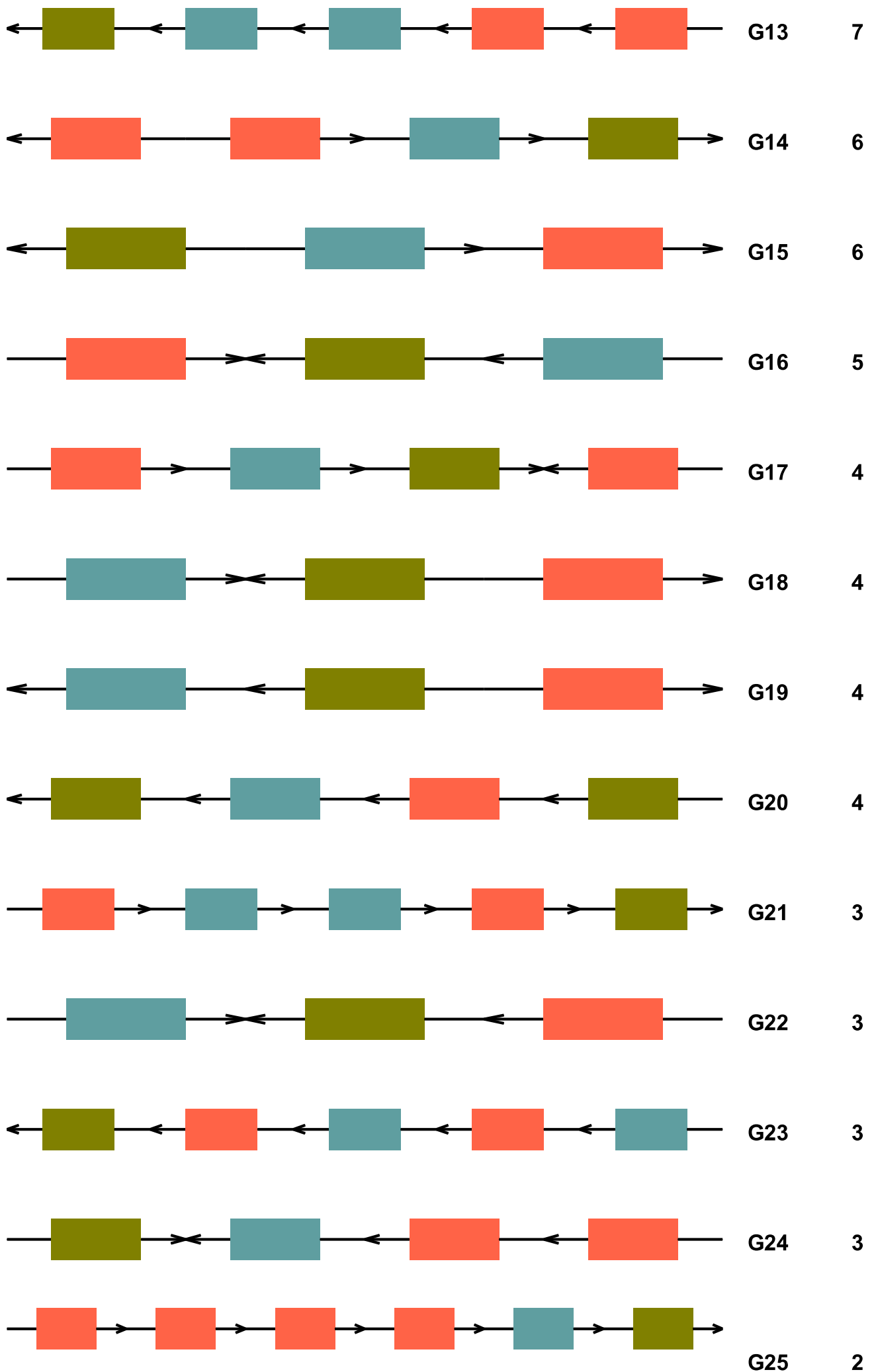

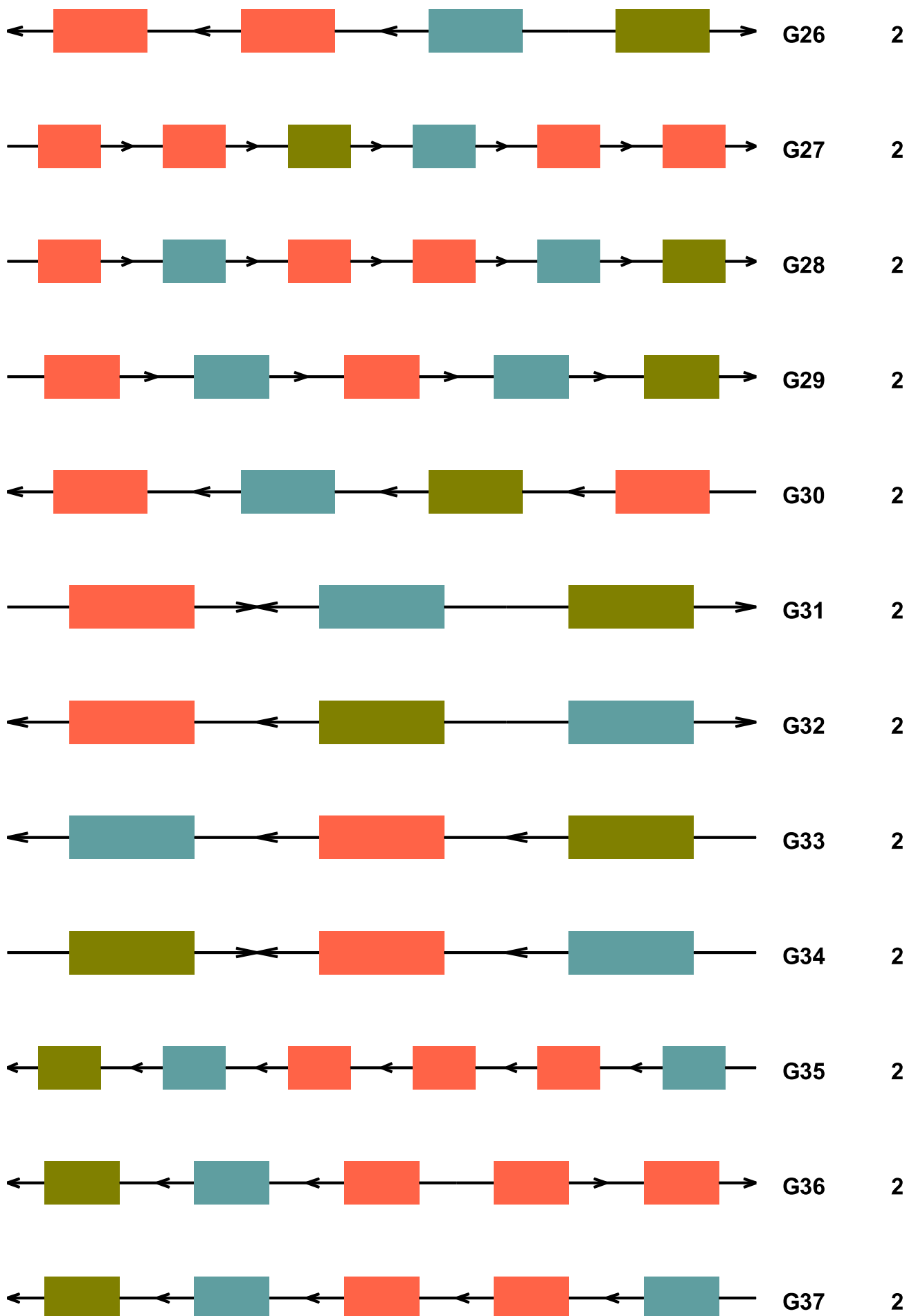

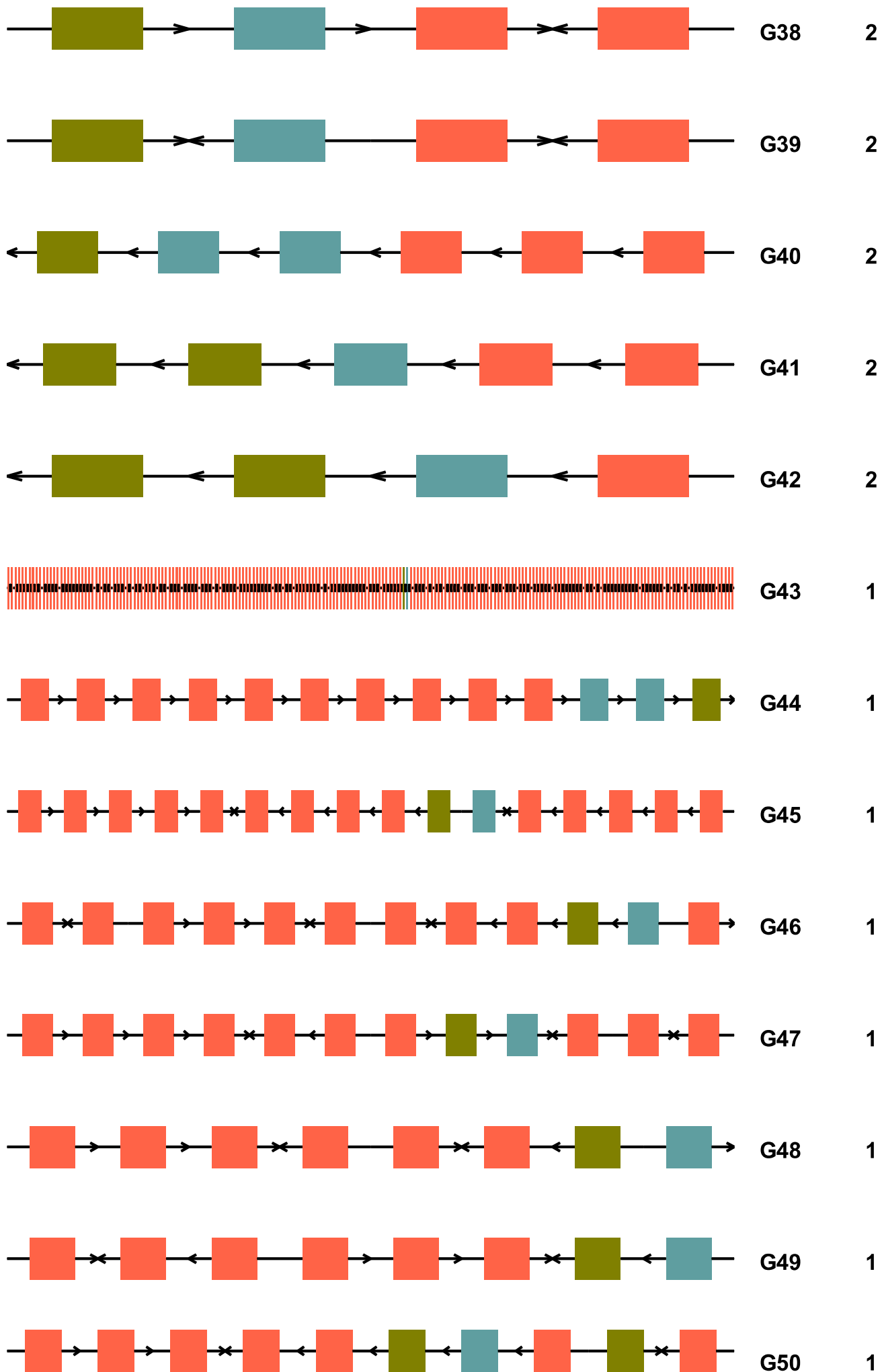

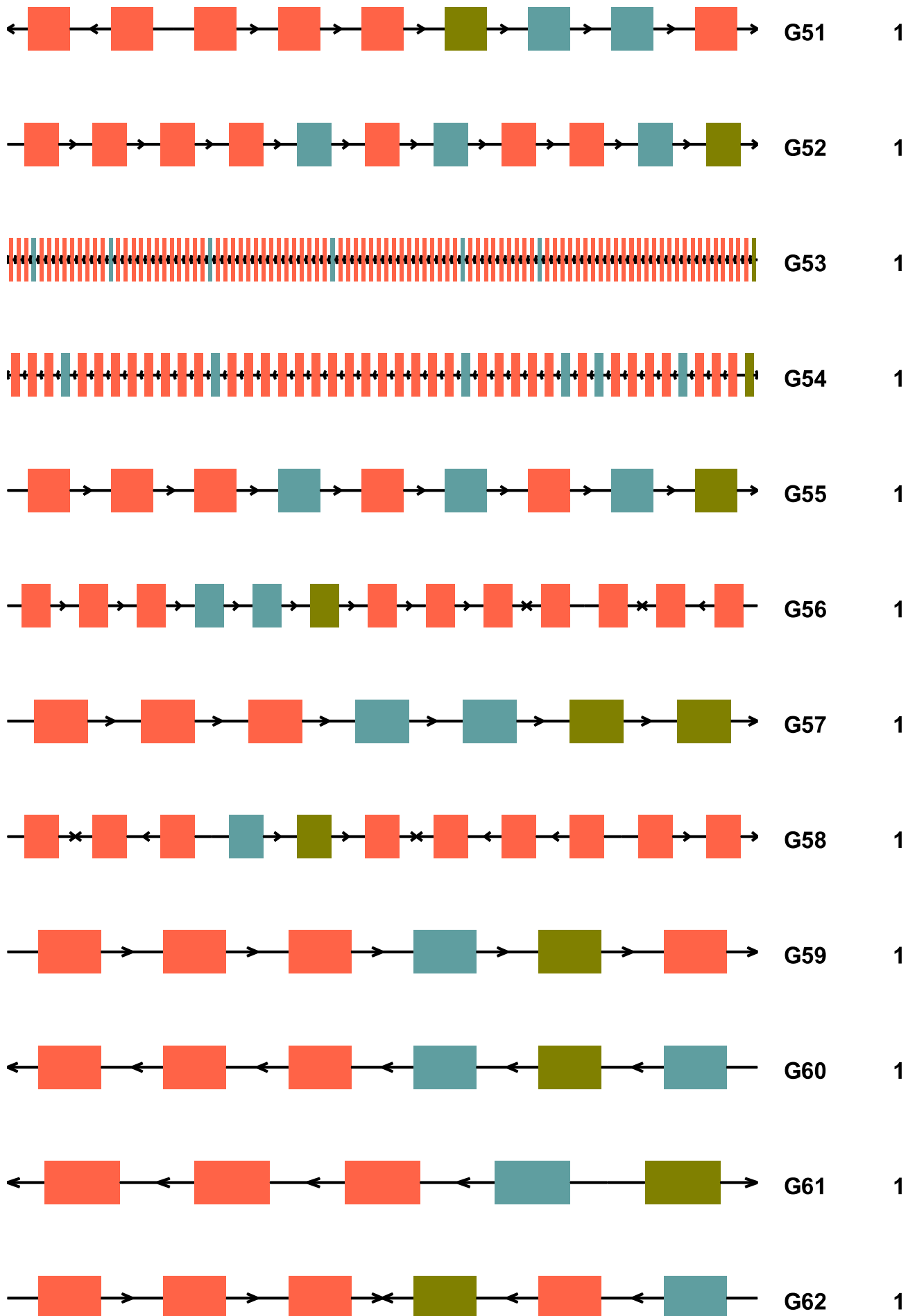

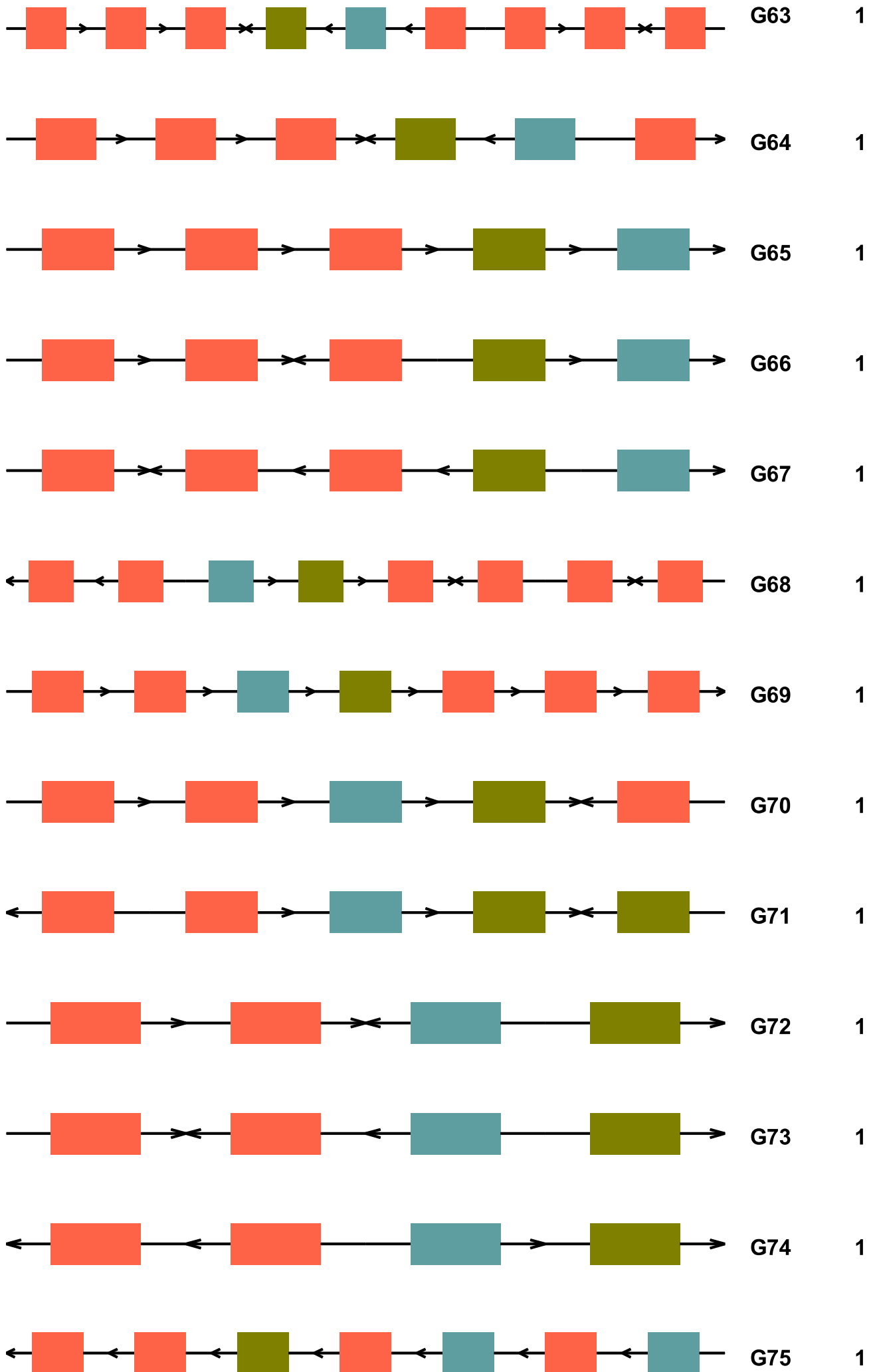

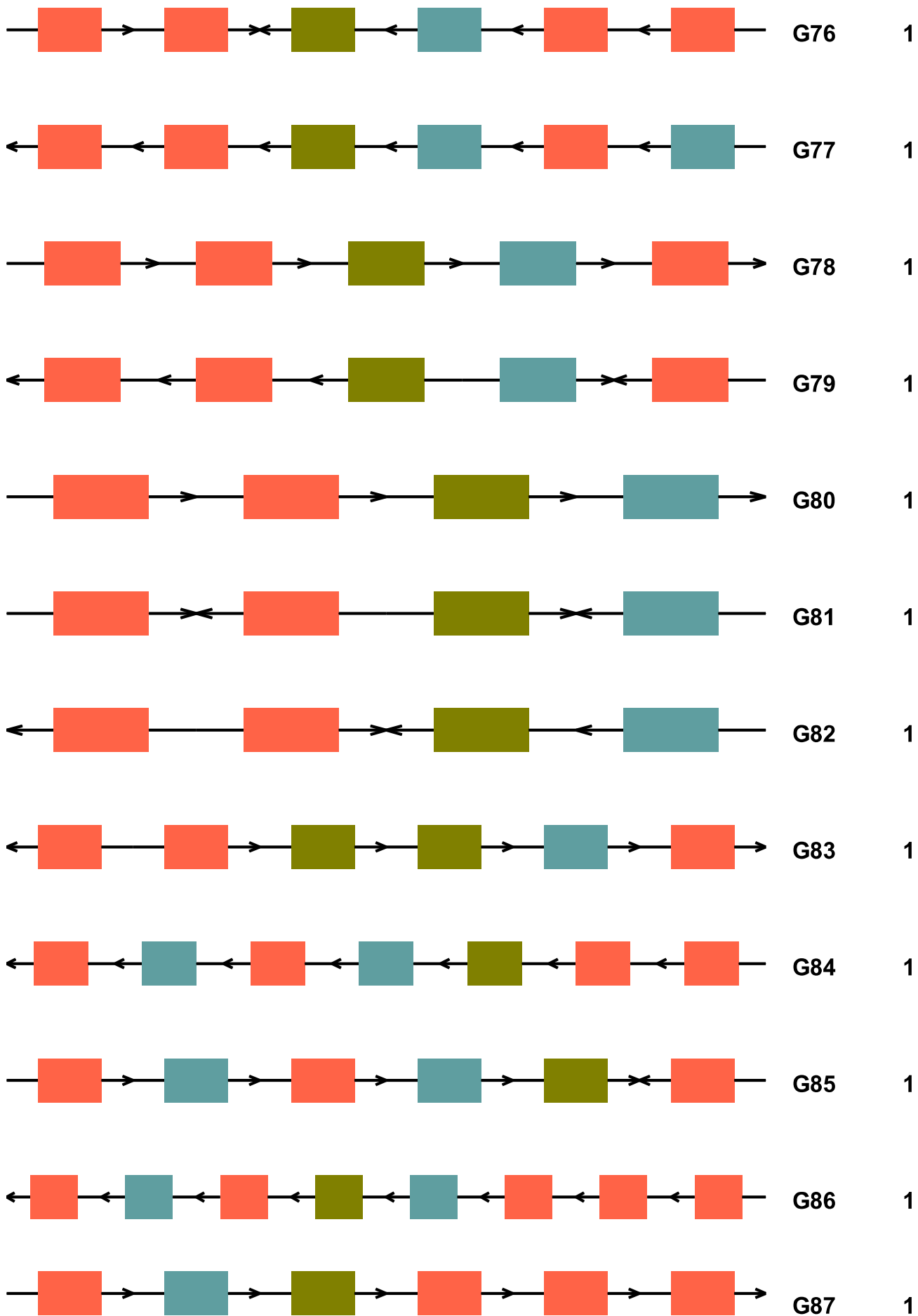

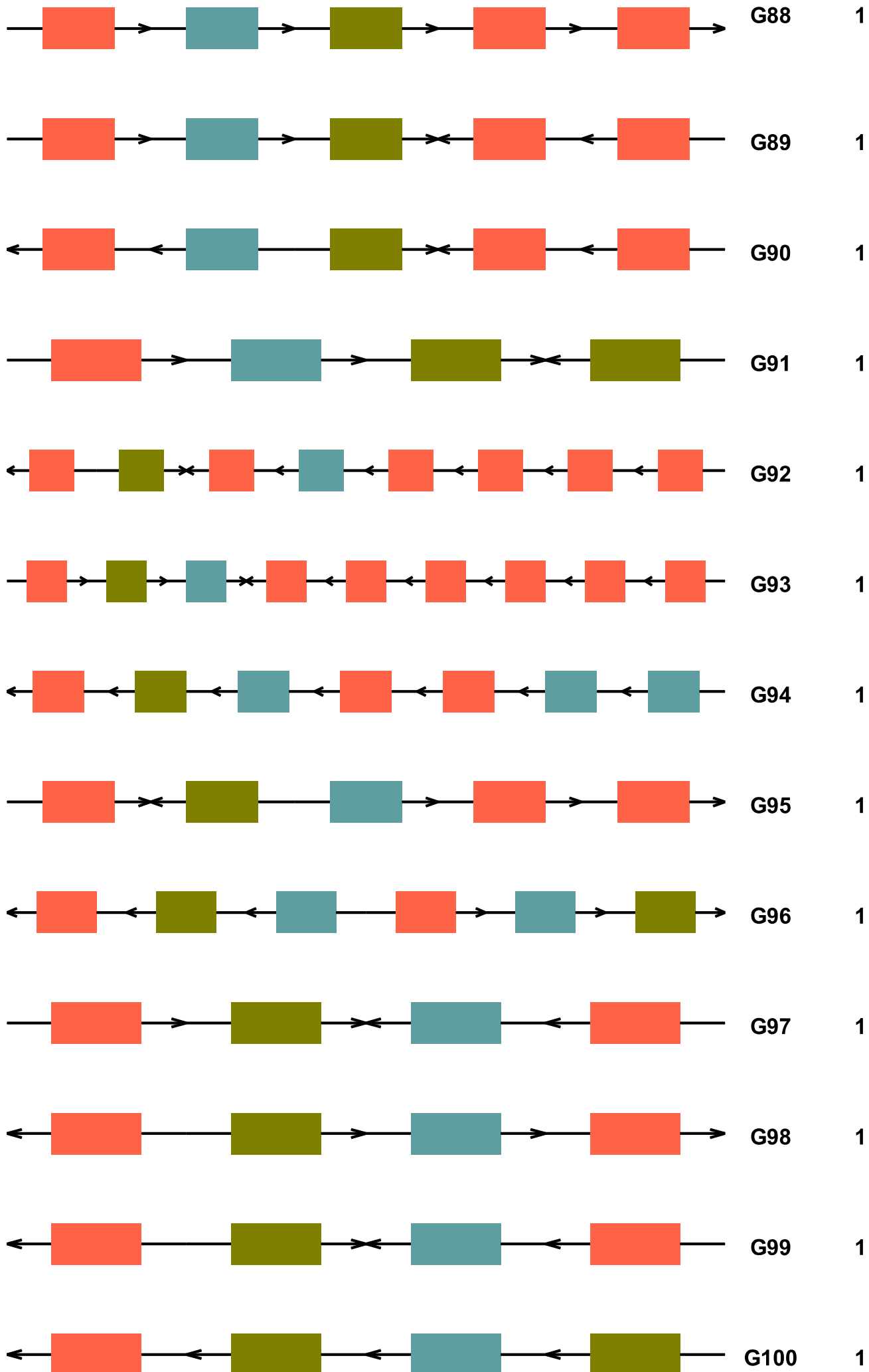

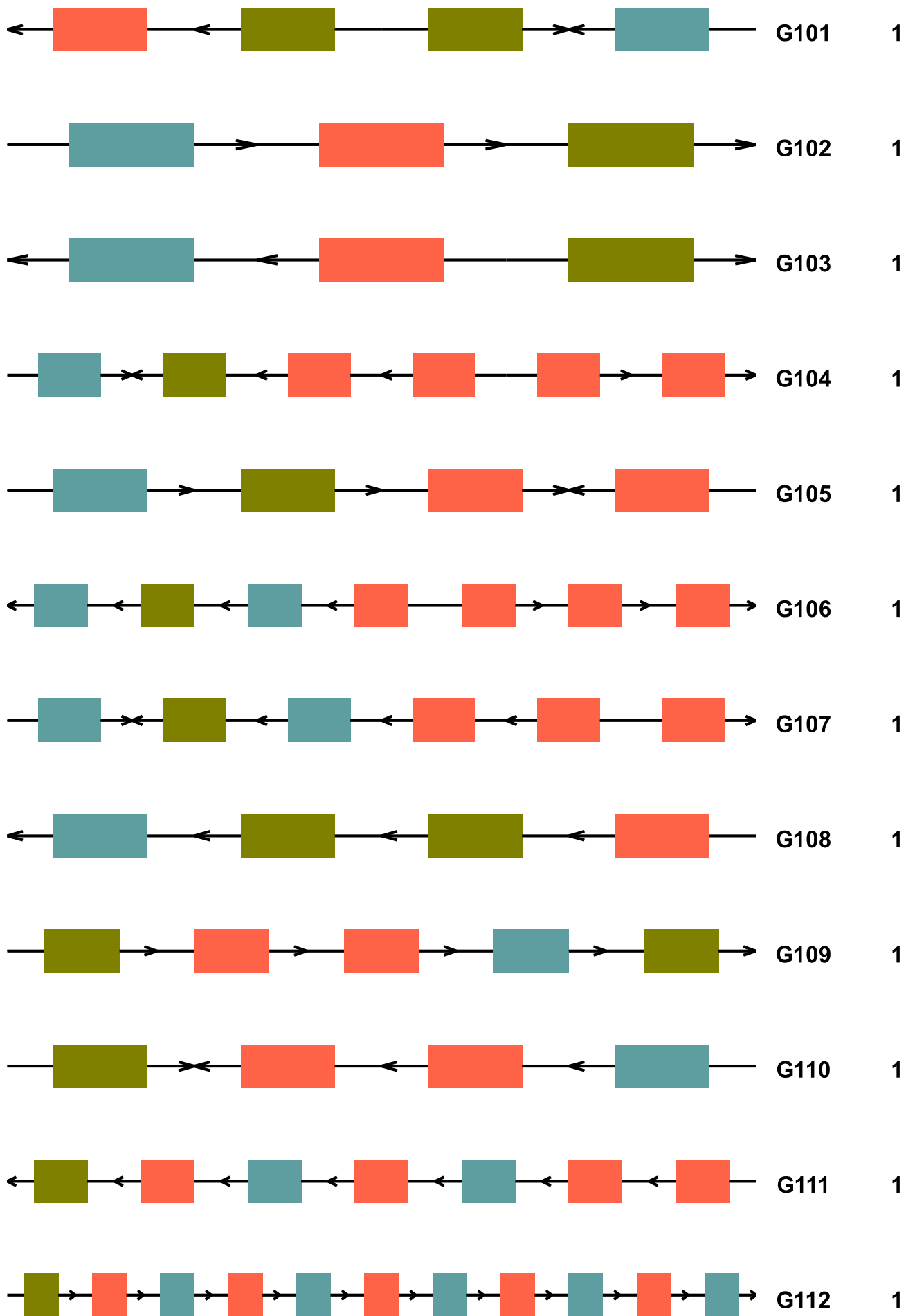

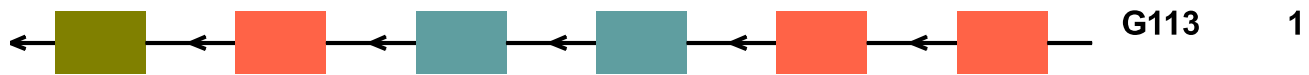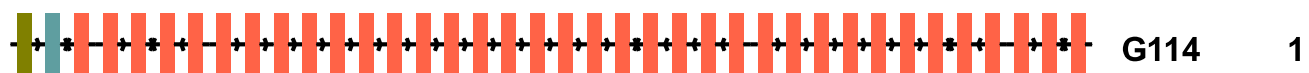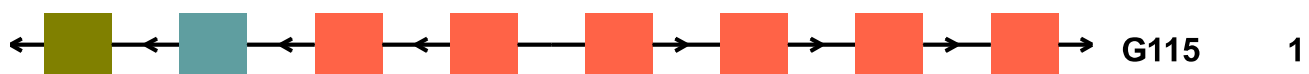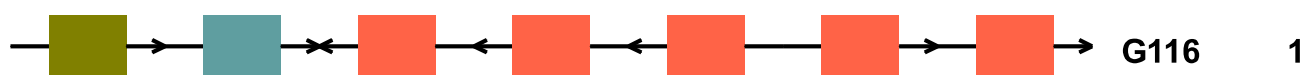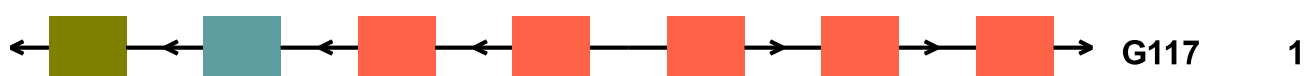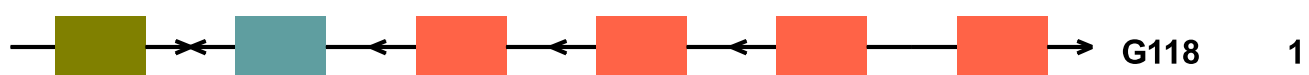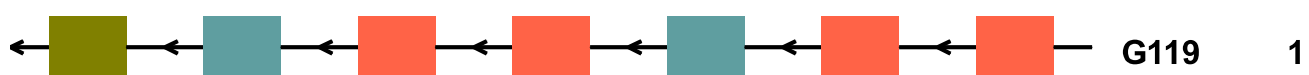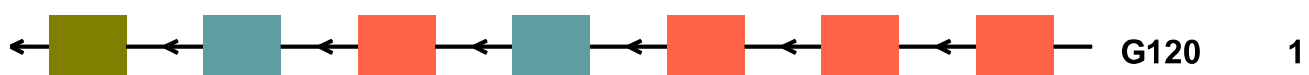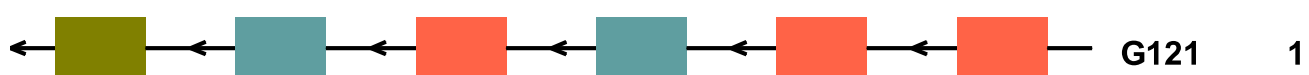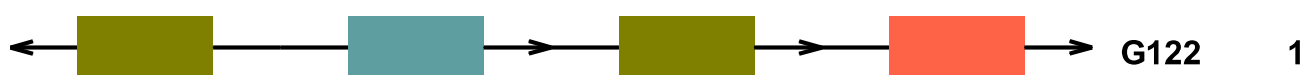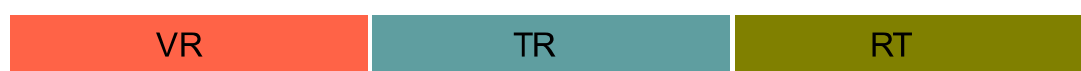

Supplement: Supplementary file 18 — Data 4. 122 cassette structures of 948 non-redundant DGRs. URL: http://cgm.sjtu.edu.cn/index/pub/software/MetaCSST/supplementary/Supplementary_Data_4.pdf (PDF 171 kb) [file 12864_2019_5951_MOESM18_ESM.pdf]
